# Fertilization and Aridity Legacies Determine Soil Microbial Necromass Persistence Across Climates

**DOI:** 10.1111/gcb.70762

**Published:** 2026-02-23

**Authors:** Li‐Xin Xu, Guang‐Hui Yu, Cong‐Qiang Liu, Georg Guggenberger

**Affiliations:** ^1^ Institute of Surface‐Earth System Science, School of Earth System Science, Tianjin Key Laboratory of Earth Critical Zone Science and Sustainable Development in Bohai Rim Tianjin University Tianjin China; ^2^ Institute of Earth System Sciences, Section Soil Science Leibniz University Hannover Germany

**Keywords:** μ‐FTIR, amino sugars, humification index, legacy effects, long‐term fertilization, necromass‐mineral interactions, reactive minerals

## Abstract

Microbial necromass‐mineral interactions are vital for soil organic carbon (C) persistence, but their response to concurrent climate and fertilization legacies in croplands remains unclear. Using six long‐term field sites (27–38 years) across a pronounced aridity gradient, we show that stabilization of microbial necromass is coupled with short‐range ordered (SRO) minerals in humid zones (aridity index, AI < 1.3). In arid zones (AI > 1.3), this correlation strongly weakens, challenging the universality of SRO‐associated C paradigms. While aridity controls mineral weathering and SRO abundance, we showed that long‐term fertilization can supersede aridity‐driven constraints on necromass C accrual. Spatial mapping of 6060 in situ μ‐FTIR spectra revealed reduced organic C transformation in outer aggregate rims under aridity, aligning with diminished microbial processing and a consequent weakening of necromass‐SRO associations. Our findings establish soil moisture as a critical regulator of fertilization‐induced ecological memory in mineral‐necromass interactions, urging climate‐tailored practices to sustain soil C resilience in water‐limited ecosystems under global aridification.

## Graphical abstract

Climate and fertilization legacies interactively regulate microbial necromass stabilization. While mineral‐mediated protection (SRO‐based) dominates in humid regions, this mechanism weakens under aridity, shifting toward climate‐driven controls.

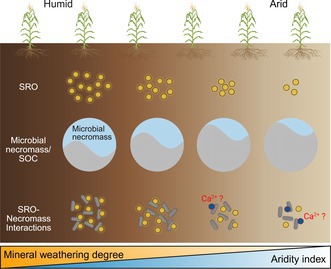

## 1 Introduction

Carbon (C) sequestration in soils represents a pivotal natural climate solution, with croplands offering considerable potential to offset anthropogenic CO_2_ emissions through restoring depleted soil organic C (SOC) stocks (Bossio et al. 2020; Lal 2004). Intensive agriculture since the Industrial Revolution has triggered a 50% loss of SOC in global croplands, compromising soil fertility and food security (Ma et al. 2023; Sanderman et al. 2017). Rebuilding stable SOC, i.e., the fraction resistant to microbial decomposition, is thus critical for sustaining agricultural productivity and enhancing climate resilience (Bai and Cotrufo 2022; Sokol and Bradford 2018). A substantial portion of this persistent organic C (OC) pool is stabilized through association with reactive pedogenic mineral surfaces, which can preserve C over centuries to millennia (Creamer et al. 2019; Sokol et al. 2022). For example, iron (Fe)‐bound OC alone accounts for 33% ± 15% of global topsoil OC (~233 Pg C), serving as a key buffer against climate‐induced C loss (Jia et al. 2024; Xiao et al. 2025). Despite its importance, the mechanisms governing the formation and persistence of mineral‐associated OC (MAOC) remain poorly resolved and are likely shaped by complex interactions among climate, land management incl. fertilization, and microbial processes (Cotrufo and Lavallee 2022; Lavallee et al. 2020).

Emerging paradigms posit microbial necromass, rather than plant residues, as the dominant precursor of stable SOC (Cotrufo et al. 2015; Miltner et al. 2012). Through the “necromass stabilization hypothesis” (Cotrufo et al. 2013; Sokol and Bradford 2018) or “mineral carbon pump” framework (Mikutta et al. 2019; Xiao et al. 2025; Xiao et al. 2023), labile plant inputs are transformed by microbes into necromass that preferentially bind to reactive minerals. This process is estimated to account for more than 50% of SOC in temperate agroecosystems (Liang et al. 2019; Wang et al. 2021). Yet, its efficacy in arid regions remains uncertain, where water scarcity limits microbial activity and mineral weathering (Mayer et al. 2023; Zhu et al. 2024). This imposes two critical constraints on necromass stabilization: (i) reduced mineral weathering limits the formation of reactive mineral surfaces for OC sorption (Kramer and Chadwick 2018; Shabtai et al. 2022), and (ii) drought suppresses microbial production of extracellular polymeric substances (EPS), thereby inhibiting mineral‐organic associations (Lehmann and Kleber 2015; Oliva et al. 2024). Furthermore, ecological memory—the legacy of past environmental or management conditions—may significantly affect microbial‐mineral interactions and SOC dynamics (Qian et al. 2025; Sebilo et al. 2013). Despite the extensive global coverage of arid croplands (~45% of terrestrial land area) (Berdugo et al. 2020), the combined influence of climate, fertilization history, and microbial necromass stabilization in these regions remains largely unexplored.

**FIGURE 1:**
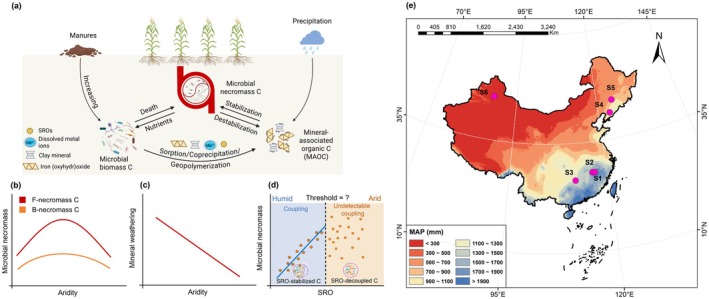
Conceptual framework of microbial necromass carbon (C) stabilization and the distribution of long‐term experimental sites. (a) Conceptual model illustrating how climate and fertilization legacies regulate mineral weathering, microbial biomass turnover, and the subsequent stabilization of necromass C in cropland soils. (b, c) Hypothetical relationships of microbial necromass C and mineral weathering in response to aridity. Microbial necromass C (including bacterial and fungal components) and mineral weathering intensity across an aridity gradient. (d) Schematic representation of the weakened association between short range ordered (SRO) minerals and microbial necromass in arid climates, reflecting a shift in stabilization dominance. (e) Geographic distribution of the six long‐term fertilization experimental sites (S1–S6) spanning humid, sub‐humid, and arid regions used to evaluate the model's applicability. MAP, mean annual precipitation. Map lines delineate study areas and do not necessarily depict accepted national boundaries.

We propose that historical fertilization practices and aridity levels jointly determine necromass stabilization by affecting necromass production and mineral reactivity (Figure 1a). Specifically, we hypothesize that (i) microbial necromass C exhibits a nonlinear (parabolic) response to increasing aridity in cropland, mirroring the patterns observed in global grasslands (Bai and Cotrufo 2022), (ii) mineral weathering decreases linearly with declining precipitation, and (iii) necromass production and its subsequent stabilization as MAOC are tightly coupled in humid systems but exhibit a weakened association under aridity, potentially due to divergent water‐limiting constraints on microbial and mineral processes (Figure 1b). To test these hypotheses, we used six long‐term (27–38 years) cropland experiments across a pronounced aridity gradient (aridity index (AI) from 0.7 to 6.1, where a higher AI reflects drier conditions), encompassing humid, sub‐humid, and arid climates (Figure 1c). These well‐managed trials minimize short‐term management variability (Rasmussen et al. 1998) and provide a unique platform to evaluate the legacy effects (Qian et al. 2025; Sebilo et al. 2013) of fertilization and aridity on microbial and mineral dynamics. We quantified microbial necromass C using amino sugar biomarkers (Zhang and Amelung 1996) and measured short‐range ordered (SRO) minerals and reactive Fe phases via selective chemical extractions (Blakemore et al. 1981; Mehra and Jackson 1958). Critically, we applied in situ synchrotron infrared spectroscopy (Lehmann et al. 2007) to assess the degradation state of organic matter (via humification indices (Hodgkins et al. 2014)) across soil aggregates. Our findings highlight soil water availability as a key regulator of fertilization‐induced ecological memory, governing SOC persistence through its control over necromass‐mineral interactions. This underscores the urgency of adapting carbon management strategies to regional climate, particularly in drought‐vulnerable agroecosystems.

## 2 Materials and Methods

### 2.1 Field Experiments and Sampling

Six long‐term cropland experiments (27–38 years) spanning an aridity gradient (aridity index, AI = 0.7–6.1, where AI is the ratio of average annual potential evapotranspiration to precipitation and a higher AI reflects drier conditions) were selected (Figure 1c and Data S1). Sites represented humid (AI < 1.3), sub‐humid (1.3 < AI < 1.5), and arid (AI > 5) climates (Spinoni et al. 2015). Climate data (Data S1) were sourced from the National Tibetan Plateau Data Center (http://data.tpdc.ac.cn) (Peng et al. 2019). Soils were classified according to the WRB system (WRB 2022) as Cambisols (Red soil) at sites S1–S3, Alisols (Brown soil) at S4, Phaeozem (Black soil) at S5, and Calcisol (Desert soil) at S6. Detailed physical and chemical properties for each soil type are provided in Table S1 and Data S1.

Three fertilization treatments were examined at each site: no fertilization (Control), mineral fertilization with nitrogen, phosphorus, and potassium (NPK), and NPK combined with manure (NPKM). Specific application rates are detailed in Data S1. Maize‐dominated rotations (five sites) and peanut (one site) were sampled post‐harvest (2017–2020). Topsoils (0–20 cm) were collected diagonally; nine cores per plot homogenized into composite samples. Each treatment at each site had two or three replicates (Data S1). Soil physicochemical properties for all sites are provided in Data S1 and Data S2.

### 2.2 Soil Carbon Property Analysis

Soil pH was measured potentiometrically using a pH electrode at a 1:2.5 soil/distilled water ratio. Soil dissolved organic carbon (DOC) was extracted using a 1:5 w/v ratio of deionized water on a horizontal shaker (170 rpm) at 25°C for 24 h, followed by centrifugation at 3000 *g* for 10 min. DOC was measured using a total organic carbon/total nitrogen analyzer (Multi N/C 3000, Analytik Jena AG, Germany). SOC and total nitrogen (TN) were quantified using an element analyzer (Vario EL CUBE, Elementar, Germany). Amino sugars as microbial biomarkers were analyzed by hydrolyzing soil using 6 M HCl at 105°C for 8 h (Zhang and Amelung 1996). In brief, 0.3 mg of nitrogen‐containing soil was subjected to hydrolysis. Following this, the samples were filtered, rotary evaporated, and lyophilized. The resulting residue was dissolved in anhydrous methanol to extract amino sugar monomers. A standard mixture of amino sugars, including glucosamine (GluN), galactosamine, mannosamine, muramic acid (MurA), myo‐inositol, and N‐methylglucamine, along with 1 mL of deionized water, was prepared and used as authentic standards. Amino sugar derivatives were identified by comparing their retention times with those of the standards. Derivatization converted the high‐boiling amino sugars into volatile aldononitrile acetate derivatives. The extracts were analyzed using a gas chromatography‐triple quadrupole mass spectrometer (EXPEC 5231 GC‐QQQ MS, Hangzhou EXPEC Technology Co. Ltd., China). Quantification was performed based on peak areas, expressed as mass per mass of soil (μg g^−1^), relative to an internal standard (myo‐inositol) added before sample pre‐purification. The fungal and bacterial necromass C were estimated using equations (Equations (1) and (2)) (Liang et al. 2019);

(1) B−necromassC=MurA×45

(2) $$F-\text{necromass}\;C=\left(\text{GluN}/179.17-2\times \text{MurA}/251.23\right)\times 179.17\times 9$$

where 45 and 9 are conversion factors; 179.17 and 251.23 are the molecular weights of GluN and MurA, respectively. Total microbial necromass C was calculated as the sum of fungal and bacterial necromass C. The ratio of microbial necromass C to SOC was used to assess the microbial contribution to SOC.

To quantify mineral‐associated organic carbon (MAOC), a density fractionation method was employed to separate mineral‐associated organic matter (MAOM) from particulate organic matter (POM) (Golchin et al. 1994). Six grams of oven‐dried soil were mixed with 30 mL of sodium polytungstate (SPT) adjusted to a density of 1.85 g cm^−3^, along with twelve ~4 mm glass beads. The mixture was shaken at 120 rpm for 18 h to disperse soil aggregates. The suspension was centrifuged at 2325 *g* for 20 min, and the supernatant—containing the light fraction, mostly consisting of POM, was collected, filtered under vacuum, rinsed with deionized water, and freeze‐dried. The remaining heavy fraction (mostly represented by MAOM) was resuspended in deionized water and centrifuged at 2325 *g* for 25 min. This washing step was repeated three times to remove residual SPT. The MAOM fraction was then freeze‐dried to a constant weight.

### 2.3 Mineral Phase and Iron‐Associated OC Analyses

Reactive Fe minerals (Fe_d_) were extracted using the dithionite‐citrate‐bicarbonate (DCB) method (Lalonde et al. 2012; Mehra and Jackson 1958). Briefly, 0.25 g soil was heated at 80°C for 1 h in a citrate‐bicarbonate buffer containing 0.25 g Na_2_S_2_O_4_. The supernatants were acidified (pH < 2 with HCl), centrifuged, and analyzed for Fe concentrations using an Inductively Coupled Plasma Optical Emission Spectrometer (ICP‐OES; 5110, Agilent, Melbourne, Australia). A control extraction, substituting sodium citrate and bicarbonate with NaCl, was conducted to correct the release of water‐soluble OC. The proportion of Fe‐bound OC (%) was calculated as follows Equation (3):

(3) $$\mathrm{Fe}-\text{bound}\;\mathrm{OC}\;\left(\%\right)=\left({\mathrm{OC}}_{\text{NaCl}}-{\mathrm{OC}}_{\mathrm{DCB}}\right)/\mathrm{SOC}\times 100$$

where OC_NaCl_ and OC_DCB_ are the OC contents in NaCl‐ and DCB‐extracted residues, respectively.

Short‐range ordered (SRO) minerals were extracted with 0.2 M acid ammonium oxalate (Blakemore et al. 1981), which selectively dissolves poorly crystalline minerals such as ferrihydrite and allophane, but not crystalline or layered silicates (Wagai et al. 2020). Oxalate‐extractable Fe and Al (Fe_o_ and Al_o_) were measured using ICP‐OES, and SRO content was calculated as Al_o_ + 1/2Fe_o_. The ratio of Fe_d_ to total Fe (Fe_d_/Fe_t_) was used to indicate the degree of mineral weathering (Doetterl et al. 2018; Jaworska et al. 2016). Total Fe (Fe_t_) was determined via microwave‐assisted digestion using a HNO_3_‐HCl‐HF acid mixture of (6: 3: 2, v/v) followed by ICP‐OES analysis. Exchangeable calcium (Ca^2+^) and magnesium (Mg^2+^) were extracted using a 70% ethanol solution of ammonium chloride, following the removal of soluble chlorides and sulfates with 70% ethanol (Chen et al. 2021), and quantified by ICP‐OES.

### 2.4 Spatially Resolved Humification Index Analysis

To evaluate the degree of organic matter degradation, we calculated a spatially resolved humification index (HI), defined as the ratio of absorbance at 1643 cm^−1^ (amide) to 1030 cm^−1^ (polysaccharide) (Hodgkins et al. 2014), using synchrotron infrared (IR) spectromicroscopy. Because humification involves the enrichment of recalcitrant moieties such as amides relative to labile polysaccharides, this micro‐(μ)FTIR‐based HI serves as a proxy for the extent of organic matter transformation (Beer et al. 2008; Broder et al. 2012; Hodgkins et al. 2014).

Thirty‐six intact soil microaggregate samples (< 250 μm) were collected from six sites, covering three fertilization treatments and two replicates pretreatment. Microaggregates were hand‐picked, placed on fiber filter paper, and maintained in a moist state for 24 h (Lehmann et al. 2007). Samples were cryo‐sectioned into 1‐μm‐thick slices at −20°C using a Cryomicrotome (Thermo Shandon Limited, UK), and then mounted onto MirrIR Low‐E microscope slides (Kevley Technologies, USA).

Fourier‐transform infrared (FTIR) spectroscopy was conducted in reflectance mode using a Thermo Nicolet 6700 spectrometer at Beamline 01B of the Shanghai Synchrotron Radiation Facility (SSRF), China (Guo et al. 2024). Specific acquisition parameters were as follows: range 4000–650 cm^−1^, aperture size 10 × 10 μm^2^, step size 1 μm; spectral resolution 4 cm^−1^, and 64 scans per pixel. A total of 6060 spatially resolved spectra were obtained, distributed by climate zones as follows: 4092 spectra from humid sites, 980 from sub‐humid sites, and 988 from arid sites; and by fertilization treatments: 2089 from control plots, 1969 from mineral fertilization, and 2002 from mineral plus manure fertilization (Data S3).

Characteristic absorption bands were assigned as follows: 3621 cm^−1^ (structural O–H in layered phyllosilicates), 2922 cm^−1^ (C–H stretching in aliphatic lipids), 1643 cm^−1^ (N–H bending in amides), and 1030 cm^−1^ (C–OH in polysaccharides) (Lehmann et al. 2007; Weng et al. 2022). Spectral data were smoothed and baseline‐corrected using OMNIC 9.0 software (Thermo Fisher Scientific, USA). The intensities of functional group peaks were spatially mapped to visualize their distributions within microaggregates.

### 2.5 Statistical Analyses

Regression analyses, i.e., linear, quadratic, generalized additive models (GAMs), were used to evaluate relationships between microbial necromass and functional group variables. The best‐fitting model was selected based on the lowest corrected Akaike information criterion (AICc), with ΔAICc > 2 indicating significant model differences. For nonlinear relationships, thresholds were identified by systematically comparing six breakpoint models: step (intercept change), segmented (slope + intercept change), segmented (slope‐only change), linear‐quadratic (M12), quadratic‐linear (M21), and quadratic‐quadratic (M22), using step sizes of 1 (FTIR variables). The optimal threshold model was determined by AICc minimization.

Geospatial context was established using GIS tools. All analyses were performed in SPSS 26, Origin 2022, and R. Group differences were assessed via Mann–Whitney U tests with Benjamini‐Hochberg *p*‐value correction (significance: *p* < 0.05). Pearson correlations (*p* < 0.05) evaluated necromass‐mineral relationships. Random Forest analysis was employed to quantify the relative importance of environmental and management predictors for microbial necromass and mineral properties. Fertilization history was quantified using continuous variables: NPK intensity (sum of N, P, and K inputs) (Figure 3 and Figure S2), individual N, P, and K fertilizer rates (Figure S3) and manure intensity (exogenous organic matter input), with control plots representing a zero‐input baseline (Tables S2 and S3). To account for edaphic influences, soil pH, DOC, C/N ratio, and texture were included in the model. Variable importance was determined using the *rfPermute* package, with significance assessed via 1000 permutations. All variables were *Z*‐score transformed to eliminate scale‐dependent bias. Synchrotron μ‐FTIR spectra were processed using OMNIC 9.0 for peak area analysis. Data represent means ± SE.

## 3 Results

### 3.1 Fertilization Outweighs Aridity in Driving Microbial Necromass Carbon Accumulation

**FIGURE 2:**
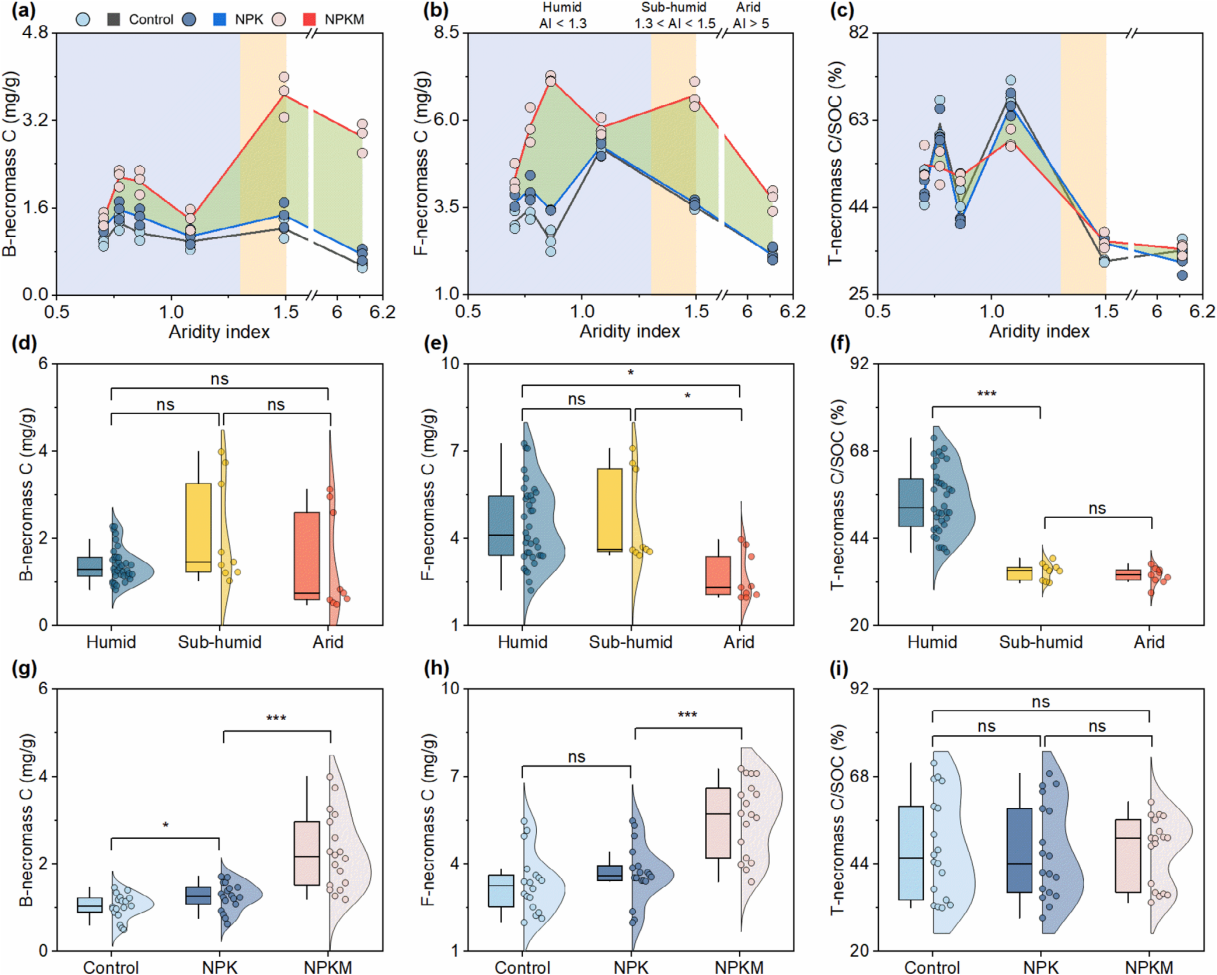
Microbial necromass carbon (C) in response to aridity index (AI) and fertilization regimes. (a–c) Changes in microbial necromass C with AI, with shaded green areas indicating increases associated with manure amendments. AI is the ratio of average annual potential evapotranspiration to precipitation and a higher AI reflects drier conditions. (d–i) Violin plots of microbial necromass C in response to climate zones (d–f) and fertilization regimes (g–i). Climatic zones are classified to humid (AI < 1.3), sub‐humid (1.3 < AI < 1.5), and arid (AI > 5). Specifically, sites S1–S4 in Figure 1 belong to humid climate, whereas sites S5 and S6 belonged to sub‐humid and arid climate, respectively. B‐necromass C, bacterial necromass C; F‐necromass C, fungal necromass C; T‐necromass C is the sum of bacterial and fungal necromass C. SOC, soil organic carbon. Control, no fertilization; NPK, mineral fertilization with nitrogen, phosphorus, and potassium; NPKM, NPK combined with manure. Significance is denoted as *p < 0.05, ***p < 0.001. Data are means ± SE.

Biomarker analyses identified distinct climatic and edaphic controls on microbial necromass C (Figure 2). Necromass accounted for 41%–68% of SOC in humid zones but declined to 32%–36% in sub‐humid/arid regions (Figure 2c), reflecting climate‐dependent stabilization. Regarding specific microbial pools, bacterial necromass C remained relatively stable across climate zones (Figure 2a,d), particularly under low C‐input conditions (unfertilized, mineral‐fertilized soils) where it showed a minimal aridity response (Figure 2a and Table S4). In contrast, fungal necromass C peaked at intermediate moisture (AI≈1.1; Figure 2b) and declined with increasing AI, particularly in the transition from sub‐humid to arid (Figure 2e). Both bacterial and fungal necromass pools correlated positively with SOC and total nitrogen (TN) (Figure S1 and Data S1), highlighting their role in organic matter formation.

Notably, manure application amplified bacterial necromass C, with the strongest effects in arid regions (130%–520% increase vs. 4%–127% in humid zones; Figure 2a and Table S4). Fungal necromass C increased gradually with manure inputs across the gradient (Figure 2b). Despite these gains, the necromass C‐to‐SOC ratio remained consistent across treatments (Figure 2f,i), indicating proportional scaling of microbial residues with SOC accrual.

**FIGURE 3:**
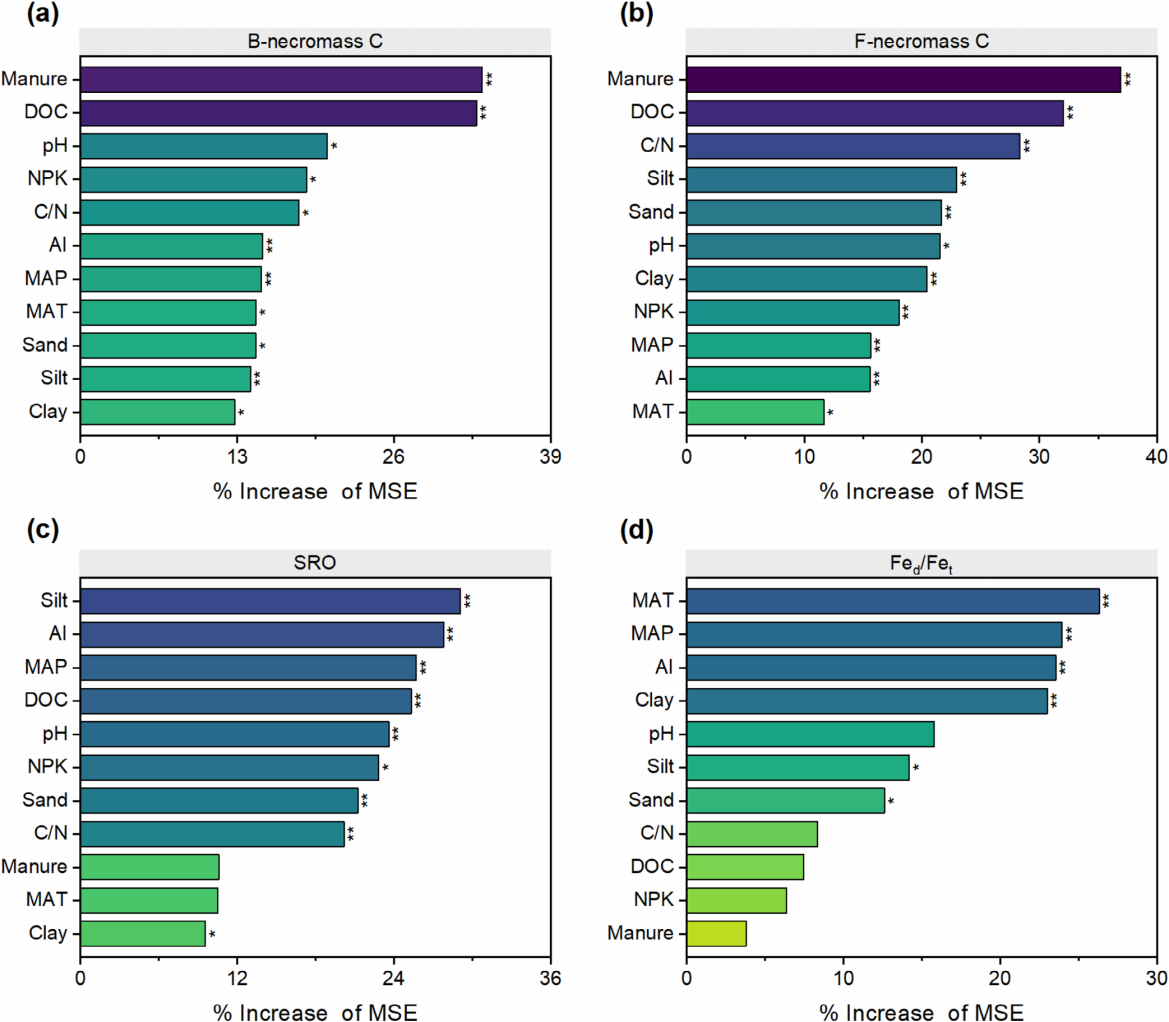
Relative importance of environmental drivers and fertilization history in predicting microbial necromass carbon and soil mineral properties. Predictor importance was determined via Random Forest analysis and expressed as the percentage increase in mean squared error (%IncMSE) following variable permutation. Panels show models for (a) bacterial necromass C, (b) fungal necromass C, (c) short‐range‐ordered (SRO) minerals, (d) dithionite‐citrate‐bicarbonate (DCB) extracted Fe (Fe_d_). AI, Aridity index; DOC, dissolved organic carbon; Fe_t_, total Fe; MAP, mean annual precipitation; MAT, mean annual temperature; NPK, mineral fertilization with nitrogen, phosphorus, and potassium. Statistical significance is indicated by asterisks: *p < 0.05, **p < 0.01 (*N* = 54).

Across fertilization regimes, bacterial necromass C remained relatively stable across climate zones (Figure 2d), whereas fungal necromass C declined with increasing AI, particularly in the transition from sub‐humid to arid (Figure 2e). Humid regions sustained higher necromass C‐to‐SOC ratios than drier ecosystems (Figure 2f), highlighting moisture as a key driver of microbial residue stabilization. Fertilization—especially manure—exerted stronger control over absolute necromass C stocks than climate (Figure 2g,h), without altering proportional SOC contributions (Figure 2i). Random forest analysis identified manure input as the primary predictor of necromass C, surpassing aridity variables (Figure 3 and Figure S2), thus reinforcing the potential of organic amendments to counteract water limitations.

Manure application and soil DOC content (Tables S1 and S2) consistently emerged as the primary predictors for both bacterial (Figure 3a) and fungal necromass C (Figure 3b). While bacterial necromass C was additionally influenced by soil pH, fungal necromass C showed a stronger dependence on the soil C/N ratio and texture (silt and sand content) (Figure 3b). In contrast, the relative contribution of necromass C to SOC was mainly controlled by climatic factors and soil texture. To further isolate the individual contributions of specific mineral nutrients, we performed a secondary Random Forest analysis by treating N, P, and K application rates as disaggregated continuous predictors (Figure S3). The results demonstrated that the relative importance of N, P, and K inputs remained marginal compared to the overriding influence of manure application and climatic factors. This reinforces the conclusion that exogenous organic matter inputs, rather than inorganic nutrient stoichiometry alone, are the primary driver of microbial necromass accrual in these systems. Furthermore, a significant interaction effect was observed for bacterial necromass C (*F* = 26.75, *p* < 0.001; Table S5), indicating that the magnitude of the fertilization response varies significantly across the aridity gradient. Manure application induced a more pronounced increase in bacterial necromass in arid regions than in humid regions. Conversely, fungal necromass C and mineral‐related variables (e.g., SRO minerals, weathering intensity) showed no significant interaction effects (*p* > 0.05; Tables S6 and S7), suggesting that fertilization and climate exert independent control on these parameters.

### 3.2 Aridity Constrains Mineral Weathering and SRO Mineral Formation

**FIGURE 4:**
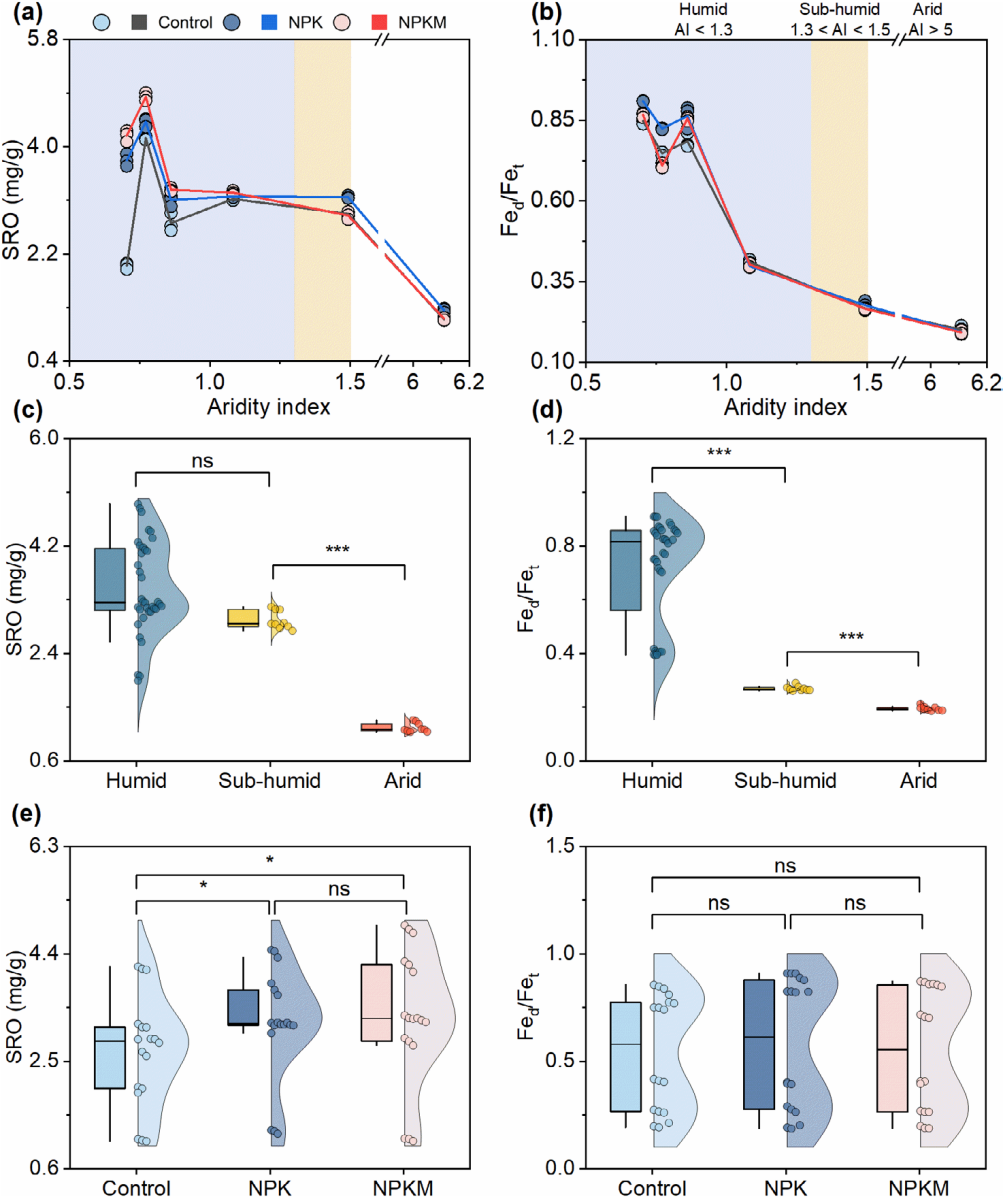
Short‐range‐ordered (SRO) mineral abundances and mineral weathering degree in response to aridity index (AI) and fertilization regimes. (a, b) Changes in SRO and Fe_d_/Fe_t_ with AI. AI is the ratio of average annual potential evapotranspiration to precipitation and a higher AI reflects drier conditions. (c–f) Violin plots of SRO mineral abundances and mineral weathering degree in response to climate zones (c, d) and fertilization regimes (e, f). Climatic zones are classified to humid (AI < 1.3), sub‐humid (1.3 < AI < 1.5), and arid (AI > 5). Specifically, sites S1–S4 in Figure 1 belonged to humid, whereas sites S5 and S6 belonged to sub‐humid and arid, respectively. Fe_d_/Fe_t_ ratio, the dithionite‐citrate‐bicarbonate (DCB) extracted Fe to total Fe ratio. Control, no fertilization; NPK, mineral fertilization with nitrogen, phosphorus, and potassium; NPKM, NPK combined with manure. Significance is denoted as *p < 0.05, ***p < 0.001. Data are means ± SE.

Increasing aridity strongly suppressed SRO mineral abundance and weathering intensity (Fe_d_/Fe_t_) (Figure 4). SRO abundance remained stable between humid and sub‐humid zones (Figure 4c), declining significantly at AI > 1.5. In contrast, mineral weathering intensity showed a significant decline with increasing aridity index (Figure 4b,d). In addition, the contents of total Fe, the reactive Fe minerals, and Fe_d‐o_ also exhibited pronounced climatic dependence (Figure S4).

Fertilization exerted a secondary influence compared to climate (Figure 4a,e). While manure application significantly increased SRO content compared to the control (Figure 4e), these effects were overshadowed by aridity constraints. Random forest analysis reinforced this hierarchy, identifying soil texture and climatic variables (MAP, aridity index, MAT) as the primary predictors of SRO abundance, weathering intensity, and other minerals, surpassing fertilization effects (Figure 3c,d). Notably, fertilization factors (Manure and NPK) ranked lowest in importance for explaining variations in mineral weathering and SRO formation, reinforcing the hierarchy where climate and texture constrain mineral evolution, while management practices dictate biological carbon accumulation.

### 3.3 Climate‐Dependent Coupling Between Microbial Necromass C and SRO Minerals

**FIGURE 5:**
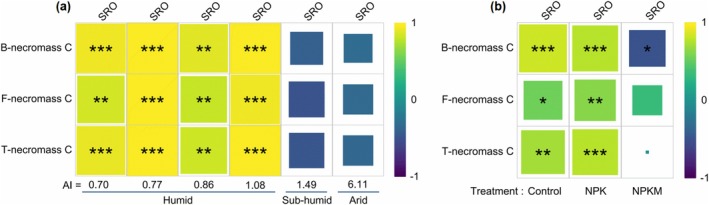
Aridity index (a) and organic fertilization (b) constraint microbial necromass carbon (C) stabilization in cropland soils. Climatic zones are classified to humid (aridity index, AI < 1.3), sub‐humid (1.3 < AI < 1.5), and arid (AI > 5). AI is the ratio of average annual potential evapotranspiration to precipitation and a higher AI reflects drier conditions. B‐necromass C, bacterial necromass C. F‐necromass C, fungal necromass C. T‐necromass C is the sum of bacterial and fungal necromass C. Control, no fertilization; NPK, mineral fertilization with nitrogen, phosphorus, and potassium; NPKM, NPK combined with manure; SRO, short‐range ordered minerals. Darker shades and bigger sizes in individual squares indicate stronger correlations between microbial necromass C and SRO. Significance is denoted as *p < 0.05, **p < 0.01, ***p < 0.001. Squares without star (*) indicate insignificant relationships (*p* > 0.05). Data are means ± SE.

**FIGURE 6:**
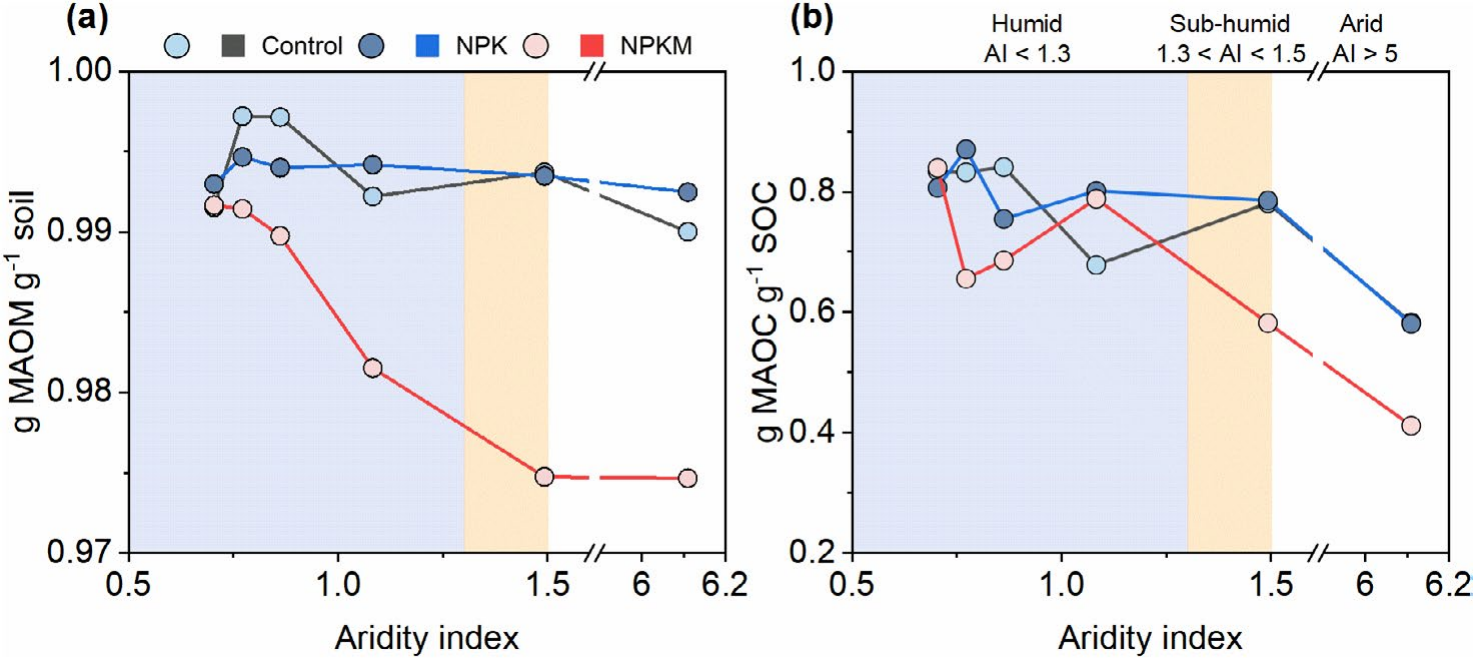
Distribution of mineral‐associated fractions across the aridity gradient. Panels show the mass proportion of (a) mineral‐associated organic matter (MAOM, g fraction kg^−1^ soil) and (b) the concentration of mineral‐associated organic carbon (MAOC, g C kg^−1^ soil) as a function of the aridity index. Control, no fertilizers; NPK, mineral fertilization with nitrogen, phosphorus, and potassium; NPKM, mineral fertilizer plus manure.

We next examined whether climate modulates the stabilization of microbial necromass C through interactions with SRO minerals. Partial correlation analysis revealed robust positive associations between necromass C (bacterial, fungal, and total) and SRO minerals in humid soils (AI < 1.3), but not in drier soils (AI > 1.3) (Figure 5). Using a density fractionation approach, we demonstrated that in humid regions, the mineral‐associated organic matter (MAOM) fraction dominated the soil mass (97%–99%; Figure 6a), with MAOC accounting for the majority of total SOC (~80%; Figure 6b). This indicates that humid climatic conditions favor the formation and accumulation of mineral‐associated organic carbon. Conversely, while the contribution of MAOC to SOC decreases and the correlation between necromass C and SRO minerals weakens in arid soils (Figure 5), these systems exhibit elevated exchangeable calcium (Ca^2+^; Figure S5). This suggests that in arid ecosystems, exchangeable calcium, rather than SRO minerals, may serve as the primary driver for organic carbon preservation.

Fertilization history further modulated these interactions: unfertilized and mineral‐fertilized soils maintained strong necromass C–SRO coupling, whereas manure application may disrupt this linkage (Figure 5). This pattern likely arose from manure‐driven necromass accumulation (Figures 2g–i and 3a,b) outpacing SRO formation (Figure 4e,f), resulting in a weak or non‐significant association between the two processes and highlighting a trade‐off between organic inputs and mineral reactivity.

### 3.4 Legacy Effects of Fertilization and Drought on Spatial Patterns of Organic Carbon Degradation in Soil Aggregates

**FIGURE 7:**
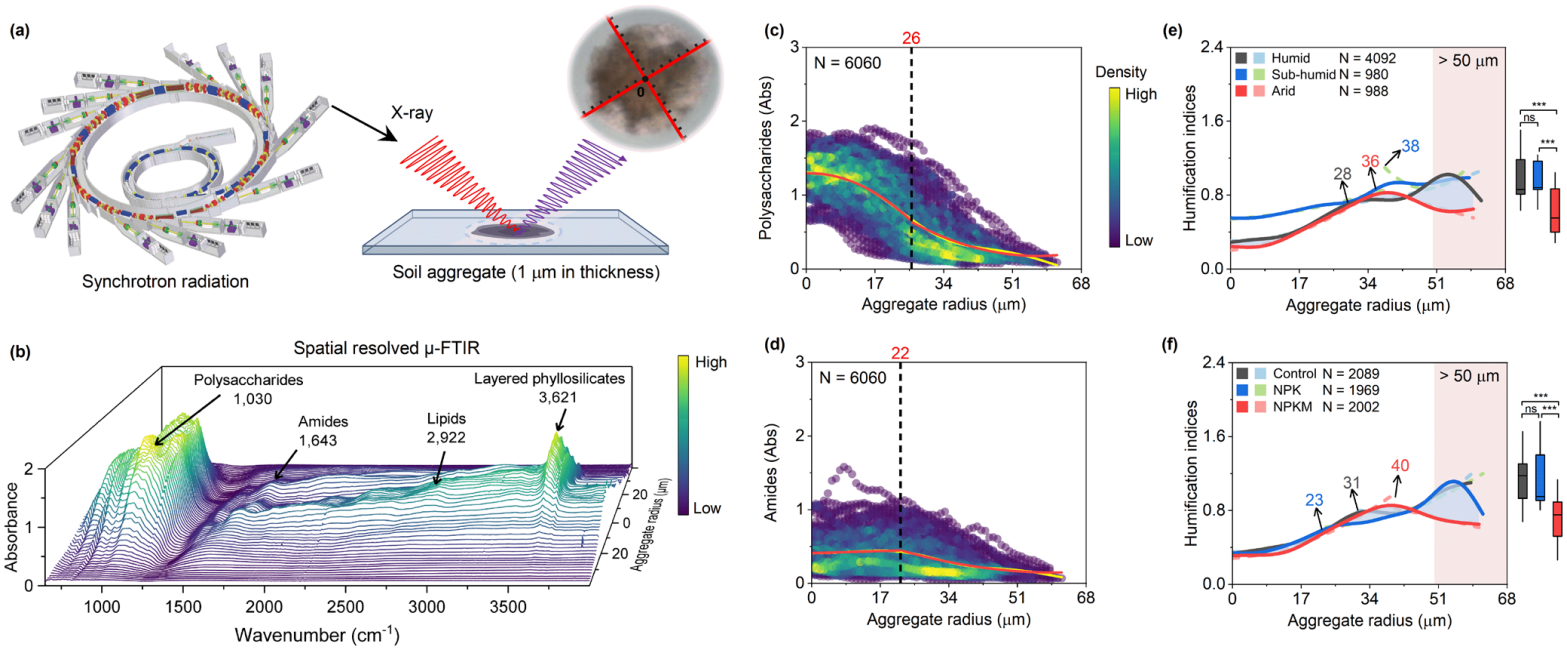
Special patterns of organic matter degradation within soil microaggregates across an aridity gradient and fertilization regimes. (a) Schematic of the in situ synchrotron μ‐FTIR spectromicroscopy platform for soil aggregate analysis. (b) Representative μ‐FTIR spectra variations across aggregate radial transects (core to rim). (c, d) Radial gradients of key organic components: Polysaccharides and amides. Smoothed trends (red lines) were fitted using generalized additive models (GAM); nonlinear fits (yellow lines) identify degradation transition thresholds (inset values). (e, f) Radial changes in humification index (HI) = amides (1643 cm^−1^)/polysaccharides (1030 cm^−1^). Shaded blue regions indicate enhanced humification associated with greater water availability and manure amendments. Solid lines show GAM fits; dashed lines indicate nonlinear thresholds. AI, aridity index. Violin plots compare HI distributions across climatic zones (Humid: AI < 1.3; Sub‐humid: 1.3 ≤ AI ≤ 5; Arid: AI > 5) and fertilization treatments (Control, No fertilization; NPK, Mineral NPK; NPKM, NPK + manure). Key infrared absorption peaks include layered phyllosilicate (3621 cm^−1^), lipids (2922 cm^−1^), amides (1643 cm^−1^), and polysaccharides (1030 cm^−1^).

Characterization of spatially resolved OC degradation within aggregates remains a major challenge. To resolve how fertilization and climate shape OC degradation patterns, we obtained 6060 spatially resolved synchrotron‐based μ‐Fourier‐transform infrared spectra (μ‐FTIR) collected from intact microaggregates (53–250 μm; Figure 7). Cryo‐ultramicrotomy preserved aggregate architecture in 1‐μm sections, enabling fine‐scale mapping of OC functional groups from core to rim (Figure 7a,b). Generalized additive models (GAMs) quantified radial decomposition gradients (Figure 7c–f and Table S8): polysaccharide absorbance declined steeply towards the rim (threshold: ~26 μm), while amides showed gentler gradients (threshold: ~22 μm; Figure 7c,d). Polysaccharide co‐localized with layered phyllosilicates (3621 cm^−1^) and lignin (1512 cm^−1^) in aggregate interiors (Figure S6).

We calculated a humification index (HI) = amide (1643 cm^−1^)/polysaccharide (1030 cm^−1^) absorbance (Figure 7e,f). HI thresholds (spatial extent of OC degradation) expanded from ~28 μm in humid soils to > 36 μm in sub‐humid/arid soils (Figure 7e,f). Notably, lower HI values in outer rims (> 50 μm radius) under aridity may indicate reduced OC degradation.

Crucially, fertilization altered these spatial patterns: mineral inputs constrained HI threshold to ~23 μm, whereas manure extended them to ~40 μm (Figure 7f), reflecting enhanced aggregate formation. Manure‐amended soils exhibited lower exterior‐rim HI values than unfertilized/mineral‐fertilized soils, indicating less advanced OC degradation.

## 4 Discussion

**FIGURE 8:**
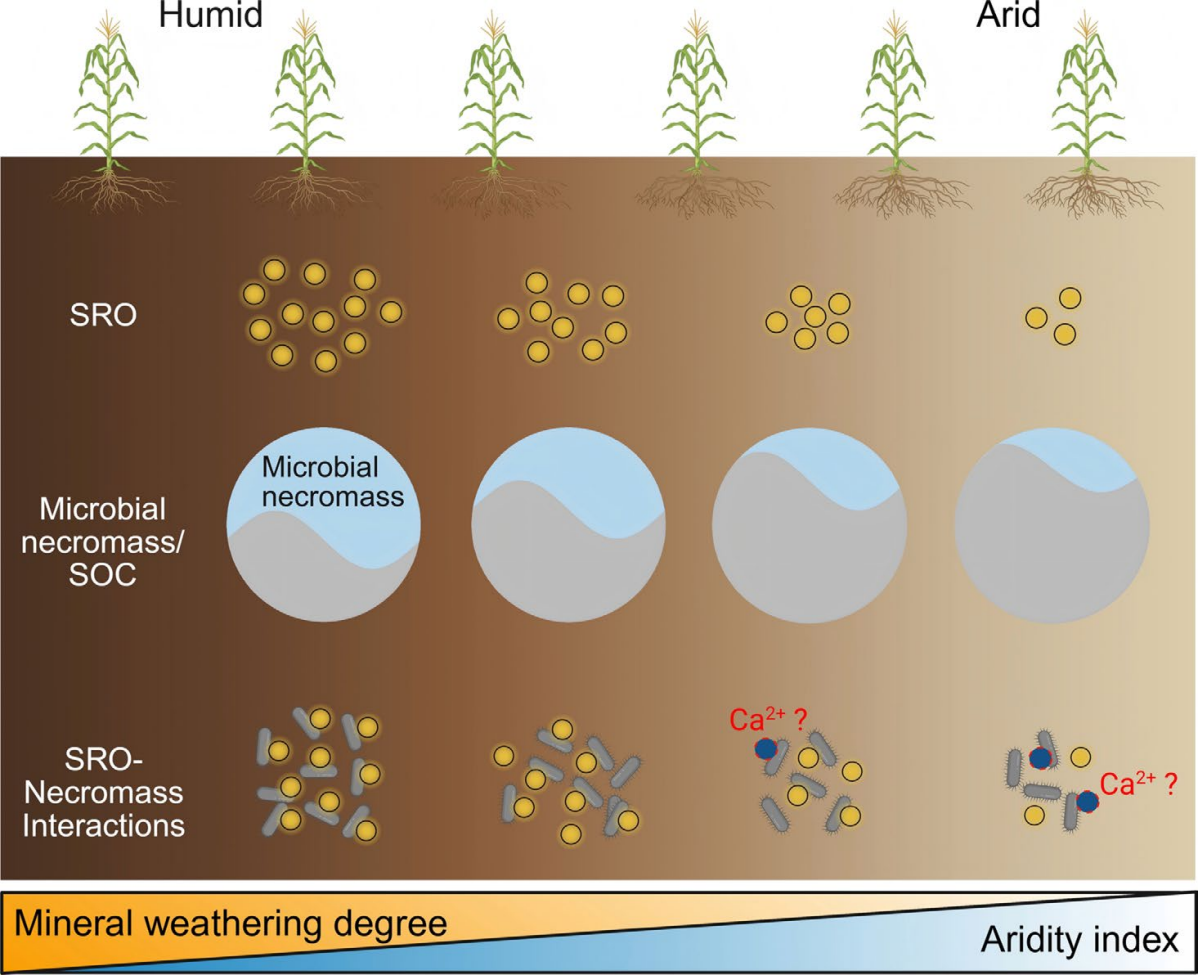
Conceptual model of microbial necromass carbon stabilization pathways across aridity and fertilization gradients. SOC, soil organic carbon; SRO, short‐range ordered minerals.

The stabilization of microbial necromass through mineral interactions, particularly with short‐range ordered (SRO) minerals, is a key mechanism for long‐term soil carbon persistence (Buckeridge, Creamer, and Whitaker 2022; Cotrufo and Lavallee 2022; Xiao et al. 2023). Yet, how climate legacies and fertilization practices jointly regulate this process in agricultural systems remains unresolved. By leveraging six long‐term field experiments across a pronounced aridity gradient, we demonstrate that drought and fertilization legacies interact to control microbial necromass stabilization by SRO minerals (Figure 8). Crucially, SRO‐mediated microbial necromass protection dominates in humid environments but becomes weakened under aridity (Figures 5 and 8). While the “mineral carbon pump” (Xiao et al. 2023; Xiao et al. 2025) provides a robust framework for understanding necromass stabilization in humid and sub‐humid systems, our results suggest its traditional reliance on SRO mineralogy as a primary driver may be context‐dependent. Under increasing aridity, the observed loss of correlation between necromass and SRO minerals does not imply a cessation of mineral‐driven stabilization, but rather a transition to alternative pathways—such as preservation mediated by Ca^2+^‐bridging—that are not currently captured by standard MCP metrics. Given that drylands cover ~45% of Earth's terrestrial surface, ~44% of which is under agriculture (Berdugo et al. 2020), and where manure is widely applied (Maillard and Angers 2014), our findings suggest the need for a climate‐sensitive expansion of the MCP framework to account for the shifting dominance of mineralogical drivers across global aridity gradients.

First, we identify soil water availability as a primary determinant of SRO mineral formation and mineral weathering (Figures 3 and 4), which constrains necromass‐SRO interactions in dry climates (Figure 5). The aridity‐driven SRO reduction (Figure 4b,d) is partially consistent with our hypothesis (ii), implying that humidity enhances Fe‐bearing mineral weathering and favors nanocrystalline phase accumulation. Aridity exerted a stronger influence on Fe_d_/Fe_t_ ratios, a well‐established proxy for mineral weathering (Doetterl et al. 2018; Jaworska et al. 2016), than on SRO mineral content (Figure 4c,d and Data S1), likely due to retention of nanocrystalline Fe phases evading SRO classification (Figure S4). This aligns with longstanding evidence that MAOC depends on mineral abundance and reactivity (Elias et al. 2024; Kramer and Chadwick 2018), both governed by climate‐driven weathering (Cotrufo and Lavallee 2022; Kramer and Chadwick 2018). Consequently, humid soils exhibited near‐exclusive dominance of MAOC over particulate organic carbon, supporting SRO minerals as critical C stabilization agents. In contrast, arid soils displayed elevated exchangeable calcium (Ca^2+^), implying that Ca^2+^‐bridging (Rowley et al. 2018) is an alternative stabilization pathway. These findings indicate that necromass stabilization is tightly mineral‐mediated in wet environments but becomes decoupled under aridity, in agreement with our hypothesis (iii). This divergence aligns with current understanding that reactive Fe/Al phases dominate C stabilization in humid soils, while base cations (Ca^2+^ and Mg^2+^) prevail in arid systems due to distinct weathering regimes (Heckman et al. 2023; Kramer and Chadwick 2018). Notably, this pattern was driven primarily by broad climatic context rather than specific irrigation regimes (sites S1–S5: rainfed; S6: drip‐irrigated since 2008; Data S1), as millennia‐scale aridity legacy likely overrides decadal‐scale irrigation. Recent studies show that MAOC concentration and persistence are tightly coupled in humid soils but decoupled in arid systems due to reduced root exudation (Heckman et al. 2023). Our findings extend this by providing field‐scale biomarker evidence that aridity disrupts necromass‐SRO stabilization, driven by drought legacies that limit moisture penetration, root dynamics, and microbial turnover—particularly in low‐productivity systems (Schiedung et al. 2023; Sebilo et al. 2013).

Second, organic amendments (manure) further weaken necromass from SRO mineral protection (Figure 5), particularly in dry regions. While manure strongly stimulates necromass production (Figures 2 and 3), it fails to enhance SRO mineral abundance or weathering (Figure 4), leaving this necromass vulnerable to decomposition in warm, dry conditions. The spatially resolved μ‐FTIR analysis (Figure 7) provides mechanistic support for this relationship. Although polysaccharides—originating from both plant and microbial residues (Kögel‐Knabner 2002)—co‐localized with layered phyllosilicates (3621 cm^−1^) in aggregate interiors (Figure S6), suggesting clay‐mediated stabilization via polyvalent cation bridging (Chenu and Stotzky 2001), the modest amide variation implied weaker protection of microbial residues. Furthermore, manure‐amended soils exhibited lower exterior‐rim HI values (Hodgkins et al. 2014) as a proxy for OC transformation (Figure 7e,f) than unfertilized soils. This indicates less advanced OC degradation and suggests that manure‐driven necromass accumulation outpaces the formation of reactive mineral surfaces. This finding from μ‐FTIR analysis implies that manure‐derived necromass may not translate to stable MAOC in drylands, necessitating climate‐tailored carbon management (Wu, Lugato, et al. 2025). Current SOC stabilization frameworks—developed largely in temperate/humid systems (Liang et al. 2019; Zhou et al. 2023)—overlook dryland constraints (Cotrufo and Lavallee 2025). Given global soil programs often extrapolate from limited climates (Cotrufo and Lavallee 2025; Sebilo et al. 2013), integrating dryland dynamics into models and policies is urgent.

Third, although site‐specific variations in nutrient stoichiometry persist—reflecting localized agricultural practices and regional management recommendations established at the inception of these long‐term experiments—our random forest analyses reveal divergent biogeochemical drivers. Specifically, manure input is the strongest predictor of necromass C, while climate factors (MAP, AI) dominate SRO mineral and weathering dynamics (Figure 3; Figures S2 and S3). This counters assumptions that climate plays a secondary role in managed systems (Cotrufo and Lavallee 2022; Paustian et al. 1997), instead highlighting that both fertilization and water availability are equally critical for necromass stabilization, especially where water scarcity intrinsically limits microbial turnover and mineral protection (Zhang et al. 2021).

Notably, fungal necromass follows a unimodal response to aridity under low C‐input conditions, aligning with our hypothesis (i). This pattern mirrors global trends observed in grasslands (Bai and Cotrufo 2022) and emphasizes a coherent climate legacy on microbial residue accumulation. The unimodal distribution suggests heightened sensitivity of fungal necromass production or stabilization to water constraints, potentially reflecting aridity‐driven nutrient limitations (Canarini et al. 2024). Furthermore, the fluctuations at the aridity threshold may reflect transient mineralization pulses during drought‐rewetting cycles—a phenomenon known as the *Birch effect* (Birch 1958), where microbial activity rapidly metabolizes previously unavailable substrates (Tang et al. 2023). While bacterial necromass remains relatively stable, this is a pattern overridden by manure amendments, with the strongest mitigation in arid zones.

This study provides a comprehensive dataset of MAOM and POM distributions across climatic and edaphic gradients, but certain limitations warrant consideration. First, while this study provides a unique evaluation of microbial necromass stabilization across a long‐term aridity gradient, certain limitations regarding site‐level heterogeneity warrant consideration. Because our sites span a broad geographic range, site‐specific variables—including reference soil groups (ranging from well weathered Cambisols and Alisols to alkaline, arid Calcisols) and distinct irrigation histories (e.g., the transition from furrow to drip irrigation at the arid site)—inevitably co‐vary with the aridity index. These pedogenic and management contrasts may partially obscure the direct influence of aridity on soil moisture and carbon cycling kinetics. Consequently, while our results highlight clear regional trends, further validation through controlled mesocosm experiments or expanded multi‐site networks that standardize soil parent material is necessary to isolate the specific mechanisms of aridity‐driven carbon “decoupling” (Di et al. 2017; Wu, Su, et al. 2025). Second, the interpretation of the μ‐FTIR‐derived humification index requires caution. While the amide band (1643 cm^−1^) is often assigned to microbial residues, the polysaccharide signal stems from both plant‐derived cellulose and microbial metabolites, which limits its diagnostic specificity (Kögel‐Knabner 2002). Ther observed spatial variations in HI are interpreted here as relative differences in the biochemical complexity of the mineral‐associated organic matter, rather than as direct evidence of process rates. Future research integrating element‐specific approaches, such as solid‐state ^13^C NMR (Hall et al. 2020) or C 1 s NEXAFS (Yu et al. 2017), along with biomarker studies (Hedges and Ertel 1982; Liang et al. 2019), will be essential to corroborate these spectral patterns with specific molecular assignments. Third, as analysis of air‐dried soils cannot fully capture in situ microbial processes, our study lacks direct microbial activity or biomass data. Future investigations using fresh soils are necessary to elucidate the microbial drivers of reactive iron formation (Li et al. 2023). Then, due to the absence of direct evidence for necromass persistence, future studies should utilize isotopic labeling (Buckeridge, Mason, et al. 2022) to precisely monitor the formation and stabilization of microbial‐derived organic matter. Finally, we acknowledge that as this study utilizes established long‐term field experiments, mineral nutrient application rates were not strictly standardized across all geographic sites. While this site‐specific variation in nutrient stoichiometry reflects regional agricultural practices, it may introduce confounding effects when comparing regional responses. Future research employing a harmonized fertilization gradient across climatic zones is necessary to further decouple the specific contributions of individual macro‐nutrients and their potential interactions with mineral weathering pathways.

Despite these limitations, to our knowledge, this is the first empirical evidence that fertilization management can supersede aridity‐driven constraints on microbial residue dynamics. Together, our findings revise current understanding of SOC persistence in dryland agroecosystems (Cotrufo and Lavallee 2025) and offer practical guidance for carbon management. In humid regions, mineral‐associated pathways play a dominant role in stabilizing microbial necromass. In contrast, in arid regions, manure amendments enhance necromass accumulation, albeit with limited SRO protection. We suggest that future research prioritize large‐scale field experiments across drylands to validate these patterns and improve the accuracy of predictive models under changing climatic conditions.

## Author Contributions

G.‐H.Y. conceived and designed the research; L.‐X.X. performed the experiments; L.‐X.X. and G.‐H.Y. analysed the data; and all authors significantly contributed to the writing of the manuscript.

## Funding

This work was supported by the National Natural Science Foundation of China, U22A20608, 32241037. Tianjin Municipal Science and Technology Bureau, 24ZYJDJC00330.

## Conflicts of Interest

The authors declare no conflicts of interest.

## Supporting information


**Figure S1:** Partial correlations between microbial necromass C and soil organic matters in cropland soils. B‐necromass C, bacterial necromass C; F‐necromass C, fungal necromass C; SOC, soil organic carbon; TN, total nitrogen. The solid lines indicate linear regressions, and the shaded areas represent 95 confidence intervals. Control, no fertilizers; NPK, mineral fertilization with nitrogen, phosphorus, and potassium; NPKM, mineral fertilizer plus manure. Different colors represent different fertilization treatments.
**Figure S2:** Relative importance of environmental factors and fertilization for predicting the microbial necromass C and minerals from random forest analysis. Variable importance is expressed as the percentage increase in mean squared error (%IncMSE) following permutation of each predictor, with higher values indicating greater importance in model prediction. T‐necromass C/SOC, total necromass C/soil organic carbon. Fe_t_, total Fe. SRO, short‐range‐ordered minerals. Fe_d_, the dithionite‐citrate‐bicarbonate (DCB) extracted Fe minerals. Fe_d‐o_, the difference between Fe_d_ and Fe_o_ subtracted. C/N: soil organic carbon/soil total nirtogen. DOC, soil dissolved organic carbon. NPK represents nitrogen, phosphorus, and potassium fertilizer application rate. Manure, organic fertilizer input. AI, aridity index; MAT, mean annual temperature; MAP, mean annual precipitation. Asterisks above bars indicate statistically significant variable importance based on permutation tests. *p < 0.05, **p < 0.01. *N* = 54.
**Figure S3:** Relative importance of environmental drivers and fertilization history in predicting microbial necromass C and soil mineral properties. Predictor importance is expressed as the percentage increase in mean squared error (%IncMSE) following permutation of each predictor, with higher values indicating greater importance in model prediction. B‐necromass C, bacterial necromass C; F‐necromass C, fungal necromass C. SRO, short‐range‐ordered minerals. Fe_t_, total Fe. Fe_d_, the dithionite‐citrate‐bicarbonate (DCB) extracted Fe minerals. T‐necromass C/SOC, total necromass C/soil organic carbon. Fe_d‐o_, the difference between Fe_d_ and Fe_o_ subtracted. C/N: soil organic carbon/soil total nirtogen. DOC, soil dissolved organic carbon. N, nitrogen fertilizer rate. P, phosphorus fertilizer rate. K, potassium fertilizer rate. MAP, mean annual precipitation. MAT, mean annual temperature. AI, aridity index. Asterisks above bars indicate statistically significant variable importance based on permutation tests. *p < 0.05, **p < 0.01. *N* = 54.
**Figure S4:** Changes in iron oxides with aridity index (AI) and fertilization regimes. (a–c) Changes in iron minerals with AI. (d–i) Violin plots of the effects of AI and fertilization on minerals. All fertilized soils are divided into three groups based on aridity to reflect soil moisture conditions. S1–S4, including YT, JX, QY, SY, represent humid soils (AI < 1.3); S5 (GZL) represent sub‐humid soils (1.3 < AI < 1.5); S6 (XJ) represent arid soils (AI > 5). Fe_t_, total Fe. Fe_d_, the dithionite‐citrate‐bicarbonate (DCB) extracted Fe minerals. Fe_d‐o_, the difference between Fe_d_ and Fe_o_ subtracted. Control, no fertilizers; NPK, mineral fertilization with nitrogen, phosphorus, and potassium; NPKM, mineral fertilizer plus manure. AI, aridity index. ***p < 0.001. Data are means ± SE. The dataset for (d–f) includes *N* = 36 (4 soils including S1–S4), 9 (1 soil including S5) and 9 (1 soil including S6) for arid soils. *N* = 18 for each treatment in (g‐i).
**Figure S5:** Changes in exchangeable elements with aridity index. The solid lines indicate linear regressions, and the shaded areas represent 95 confidence intervals. AI, aridity index. SIC, soil inorganic carbon. Control, no fertilizers; NPK, mineral fertilization with nitrogen, phosphorus, and potassium; NPKM, mineral fertilizer plus manure. Different colors represent different sites and shapes represent fertilization treatments. *N* = 54.
**Figure S6:** Changes in layered phyllosilicates with aggregate radius (a) and correlation between lignin and polysaccharides (b). Key infrared absorption peaks include layered phyllosilicate (3621 cm^−1^), lignin (1512 cm^−1^), and polysaccharides (1030 cm^−1^). The radius threshold of layered phyllosilicates is identified as 27 μm. Once this radius was reached, small increases in radius led to drastic decline in the layered phyllosilicates abundance.
**Table S1:** Changes in soil pH and DOC in response to fertilization regimes across six long‐term (27–38 years) fertilization sites^a^.
**Table S2:** Application rates of fertilizers used in RF models.
**Table S3:** Nutrient content of manure across the six long‐term experimental sites^a^.
**Table S4:** Variations in microbial necromass C for NPK and NPKM treatments compared to Control (%)^a^.
**Table S5:** Effects of fertilization and aridity index on microbial necromass C and minerals^a^.
**Table S6:** Simple effects of B‐necromass C under different aridity index and fertilization treatments^a^.
**Table S7:** Post hoc pairwise comparisons for significant main effects detected by two‐way ANOVA^a^.
**Table S8:** Best models for each variable.

**Data S1:** gcb70762‐sup‐0002‐DataS1.xlsx.

## Data Availability

All data used in this study are available in the figshare repository (https://figshare.com/) at https://doi.org/10.6084/m9.figshare.31236544, and are also provided as Supporting Information files S1–S2. Additional data that support the findings of this study are available from G.‐H.Y. upon request.
