# Supplementary material for: Fertilization and Aridity Legacies Determine Soil Microbial Necromass Persistence Across Climates
Source: Glob Chang Biol. 2026 Feb 23;32(2):e70762. doi: 10.1111/gcb.70762 (PMC12929702; doi:10.1111/gcb.70762)
Supplement: Supplementary file 1 — Figure S1: Partial correlations between microbial necromass C and soil organic matters in cropland soils. B‐necromass C, bacterial necromass C; F‐necromass C, fungal necromass C; SOC, soil organic carbon; TN, total nitrogen. The solid lines indicate linear regressions, and the shaded areas represent 95 confidence intervals. Control, no fertilizers; NPK, mineral fertilization with nitrogen, phosphorus, and potassium; NPKM, mineral fertilizer plus manure. Different colors represent different fertilization treatments. Figure S2: Relative importance of environmental factors and fertilization for predicting the microbial necromass C and minerals from random forest analysis. Variable importance is expressed as the percentage increase in mean squared error (%IncMSE) following permutation of each predictor, with higher values indicating greater importance in model prediction. T‐necromass C/SOC, total necromass C/soil organic carbon. Fet, total Fe. SRO, short‐range‐ordered minerals. Fed, the dithionite‐citrate‐bicarbonate (DCB) extracted Fe minerals. Fed‐o, the difference between Fed and Feo subtracted. C/N: soil organic carbon/soil total nirtogen. DOC, soil dissolved organic carbon. NPK represents nitrogen, phosphorus, and potassium fertilizer application rate. Manure, organic fertilizer input. AI, aridity index; MAT, mean annual temperature; MAP, mean annual precipitation. Asterisks above bars indicate statistically significant variable importance based on permutation tests. *p < 0.05, **p < 0.01. N = 54. Figure S3: Relative importance of environmental drivers and fertilization history in predicting microbial necromass C and soil mineral properties. Predictor importance is expressed as the percentage increase in mean squared error (%IncMSE) following permutation of each predictor, with higher values indicating greater importance in model prediction. B‐necromass C, bacterial necromass C; F‐necromass C, fungal necromass C. SRO, short‐range‐ordered minerals. Fet, total Fe. Fe [file GCB-32-e70762-s001.docx]

Supporting Information for

**Fertilization and aridity legacies determine soil microbial necromass persistence across climates**

Li-Xin Xu^1^, Guang-Hui Yu^1*^, Cong-Qiang Liu^1^, Georg Guggenberger^2*^

^1^Institute of Surface-Earth System Science, School of Earth System Science, Tianjin Key Laboratory of Earth Critical Zone Science and Sustainable Development in Bohai Rim, Tianjin University, Tianjin 300072, China

^2^Institute of Earth System Sciences, Section Soil Sciences, Leibniz University 30419 Hannover, Hannover, Germany^*^ Corresponding Author.

Email: yuguanghui@tju.edu.cn (G.Y.); guggenberger@ifbk.uni-hannover.de (G.G.)

**This supporting information includes:**

Supporting Materials

Supporting Methods

Figures S1 to S6

Tables S1 to S8

Datasets S1-S3

**Supporting Materials**

***Site S1 (Yingtan, Jiangxi Province), humid***

Site S1 is located at the Red Soil Ecological Experimental Station of the Chinese Academy of Sciences (28°15′N, 116°55′E) and was established in 1988 (Chen et al., 2018). The site experiences a subtropical monsoon climate with a mean annual temperature of 17.6°C, annual precipitation of 1795 mm, annual evaporation of 1318 mm, 258 frost-free days, and 1739 sunshine hours. Fertilizer application rates are provided in Supplementary Data1. Briefly, nitrogen, phosphorus, and potassium were applied as urea, superphosphate, and potassium chloride, respectively. A randomized complete block design with three replicates was used, with each plot measuring 34.6 m^2^. Cement barriers were installed between plots to prevent lateral movement of water and nutrients. Initially, a peanut–rapeseed rotation was practiced until 1995, after which it shifted to peanut monoculture with winter fallow.

***Site S2 (Jinxian, Jiangxi Province), humid***

Site S2 is situated at the Red Soil Research Institute in Jinxian (28°35′N, 116°17′E), established in 1986 (Huang, Peng, Huang, & Zhang, 2010; Liu et al., 2016; G.-H. Yu & Liu, 2022). The region has a mean annual temperature of 17.2°C, annual precipitation of 1537 mm, evaporation of 1150 mm, 259 frost-free days, and 1950 sunshine hours. Fertilizer inputs (urea, superphosphate, and potassium chloride) were applied using a randomized complete block design with three replicates and plot sizes of 22.2 m^2^. Cement barriers were used to isolate plots. The site is rainfed and follows an early maize–late maize rotation with winter fallow.

***Site S3 (Qiyang, Hunan Province), humid***

Site S3 is located at the Red Soil Experimental Station of the Chinese Academy of Agricultural Sciences (26°45′N, 111°52′E), which was established in 1990 (G. Yu et al., 2017; Zhang, Wang, Xu, & Fan, 2009). The site has a mean annual temperature of 18°C, annual precipitation of 1255 mm, evaporation of 1470 mm, 300 frost-free days, and 1610 sunshine hours. Fertilizers were applied as urea, superphosphate, and potassium chloride. A randomized complete block design with two replicates was used, with plots measuring 200 m^2^. Cement barriers separated the plots. The field is rainfed, and the cropping system follows a maize–wheat rotation.

***Site S4 (Shenyang, Liaoning Province), humid***

Site S4, established in 1979, is located at Shenyang Agricultural University (40°48′N, 123°33′E) (Hua et al., 2020; Luo et al., 2015). The site has a mean annual temperature of 8°C, annual precipitation of 684 mm, evaporation of 1436 mm, 148–180 frost-free days, and 2373 sunshine hours. Fertilizers included urea, superphosphate, and potassium sulfate. A randomized complete block design was implemented with three replicates, and each plot covered 2 m^2^. Cement barriers were used to isolate plots. The field is rainfed, and the cropping system consists of a soybean–maize–maize rotation per year.

***Site S5 (Gongzhuling, Jilin Province), sub-humid***

Site S5 is located at the National Long-Term Monitoring Base for Soil Fertility and Fertilizer Efficiency (43°30′N, 124°48′E), established in 1989 (Dou, He, Cheng, & Zhou, 2016; Song et al., 2015). The region has a mean annual temperature of 5.6 °C, annual precipitation of 562 mm, evaporation ranging from 1400 mm, 130 frost-free days, and 2710 sunshine hours. Fertilizers were applied as urea, triple superphosphate, and muriate of potash. The experimental design was a randomized complete block with three replicates and plot sizes of 130 m^2^. Cement barriers were used to prevent cross-contamination. The site is rainfed, and maize is grown in monoculture.

***Site S6 (Urumqi, Xinjiang Province), arid***

Site S6 is located at the National Modern Agricultural Science and Technology Demonstration Park of the Xinjiang Academy of Agricultural Sciences, ~25 km north of Urumqi (43°95′N, 87°46′E), and was established in 1990 (Xu, Liu, & Wang, 2016). The site experiences a continental arid climate with a mean annual temperature of 7.7°C, annual precipitation of 310 mm, evaporation of 2570 mm, 156 frost-free days, and 2590 sunshine hours (Lal, 2004). Urea, diammonium phosphate, and potassium sulfate were applied as fertilizers. The experiment was conducted in a randomized complete block design with large plots (468 m^2^), and each treatment was subdivided into three sampling areas to account for the lack of formal replication. Cement barriers were installed between plots. The field transitioned from furrow irrigation (1990–2008) to drip irrigation (since 2008). The cropping system evolved from maize–spring wheat–winter wheat (1990–1999) to cotton monoculture (1999), back to maize–spring wheat–winter wheat (2000–2008), and since 2009, has followed a cotton–maize–winter wheat rotation.

**Supporting Methods**

To assess the relative and interactive effects of fertilization and climate, two-way analysis of variance (ANOVA) was performed with fertilization and AI as fixed factors (Table S5). When significant interaction effects were detected, simple effects were examined using pairwise comparisons based on estimated marginal means (Table S6). For variables showing significant main effects without significant interactions, post hoc pairwise comparisons were conducted to evaluate differences among factor levels (Table S7). To control for multiple comparisons, *p*-values were adjusted using the Bonferroni correction.


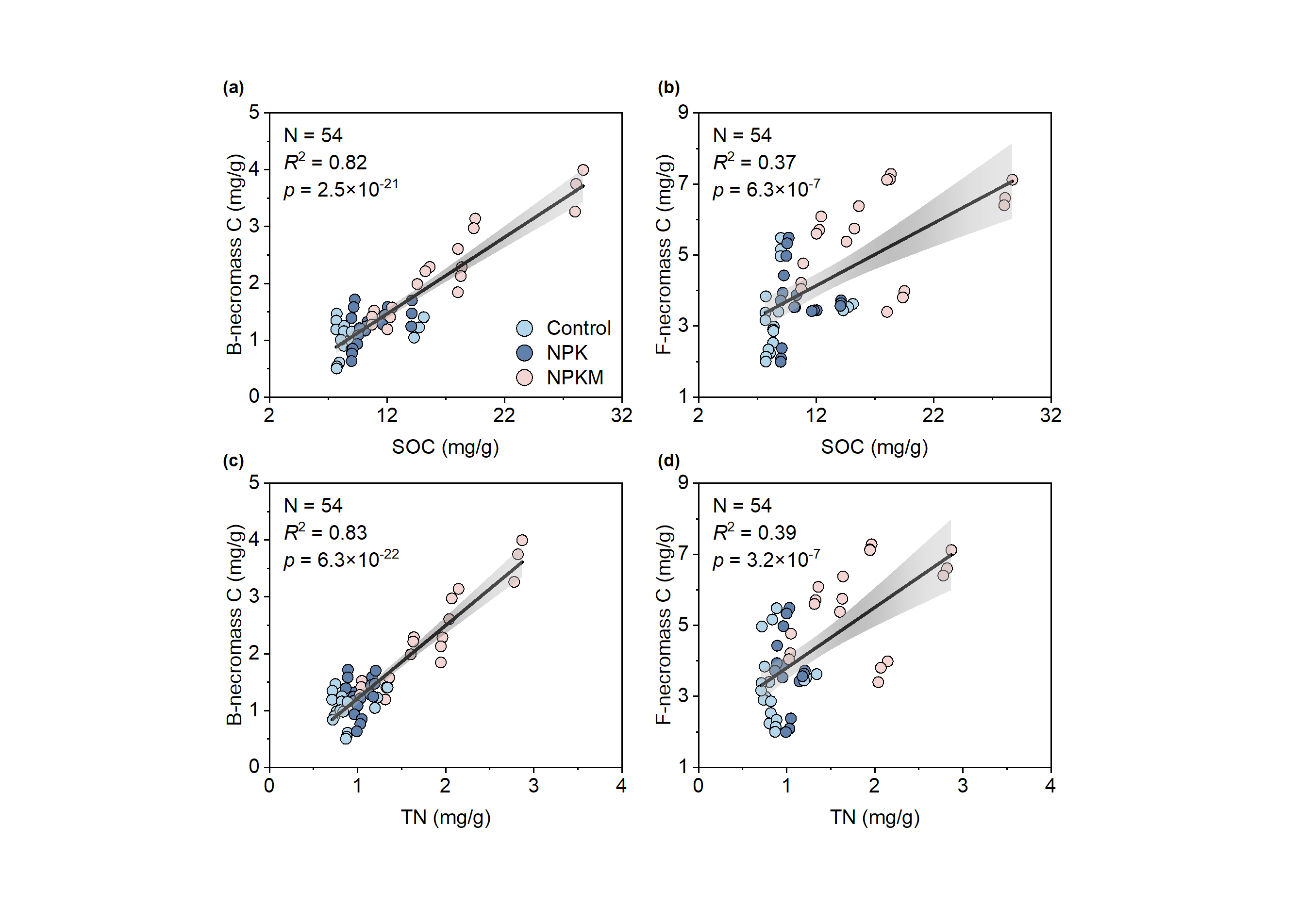


**Fig. S1.** **Partial correlations between microbial necromass C and soil organic matters in cropland soils.** B-necromass C, bacterial necromass C. F-necromass C, fungal necromass C. SOC, soil organic carbon. TN, total nitrogen. The solid lines indicate linear regressions, and the shaded areas represent 95 confidence intervals. Control, no fertilizers; NPK, mineral fertilization with nitrogen, phosphorus, and potassium; NPKM, mineral fertilizer plus manure. Different colors represent different fertilization treatments.


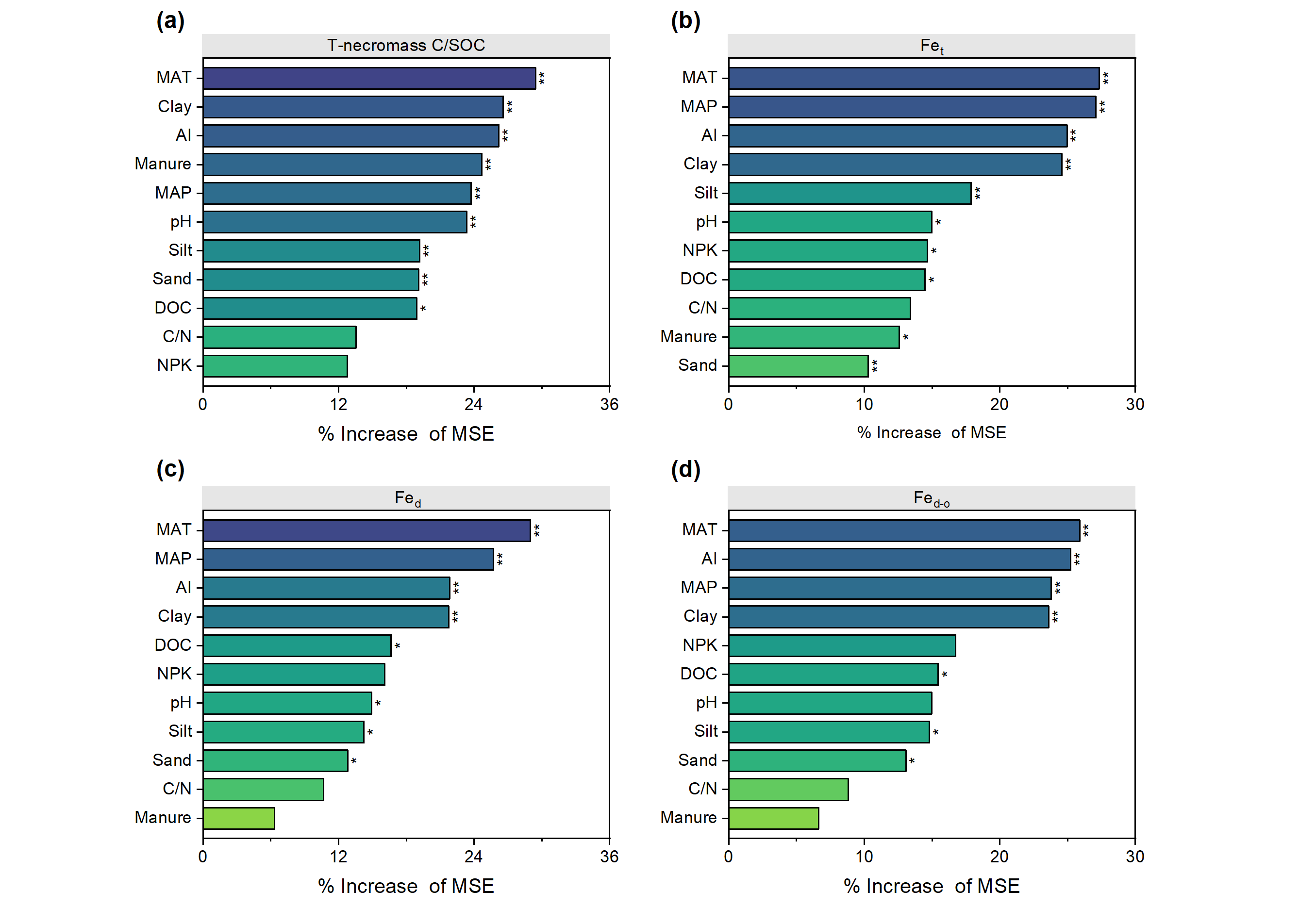


**Fig. S2. Relative importance of environmental factors and fertilization for predicting the microbial necromass C and minerals from random forest analysis.** Variable importance is expressed as the percentage increase in mean squared error (%IncMSE) following permutation of each predictor, with higher values indicating greater importance in model prediction. T-necromass C/SOC, total necromass C/ soil organic carbon. Fe_t_, total Fe. SRO, short-range-ordered minerals. Fe_d_, the dithionite-citrate-bicarbonate (DCB) extracted Fe minerals. Fe_d-o_, the difference between Fe_d_ and Fe_o_ subtracted. C/N: soil organic carbon/soil total nirtogen. DOC, soil dissolved organic carbon. NPK represents nitrogen, phosphorus, and potassium fertilizer application rate. Manure, organic fertilizer input. MAP, mean annual precipitation. MAT, mean annual temperature. AI, aridity index. Asterisks above bars indicate statistically significant variable importance based on permutation tests. **p* < 0.05, ***p* < 0.01. N = 54.


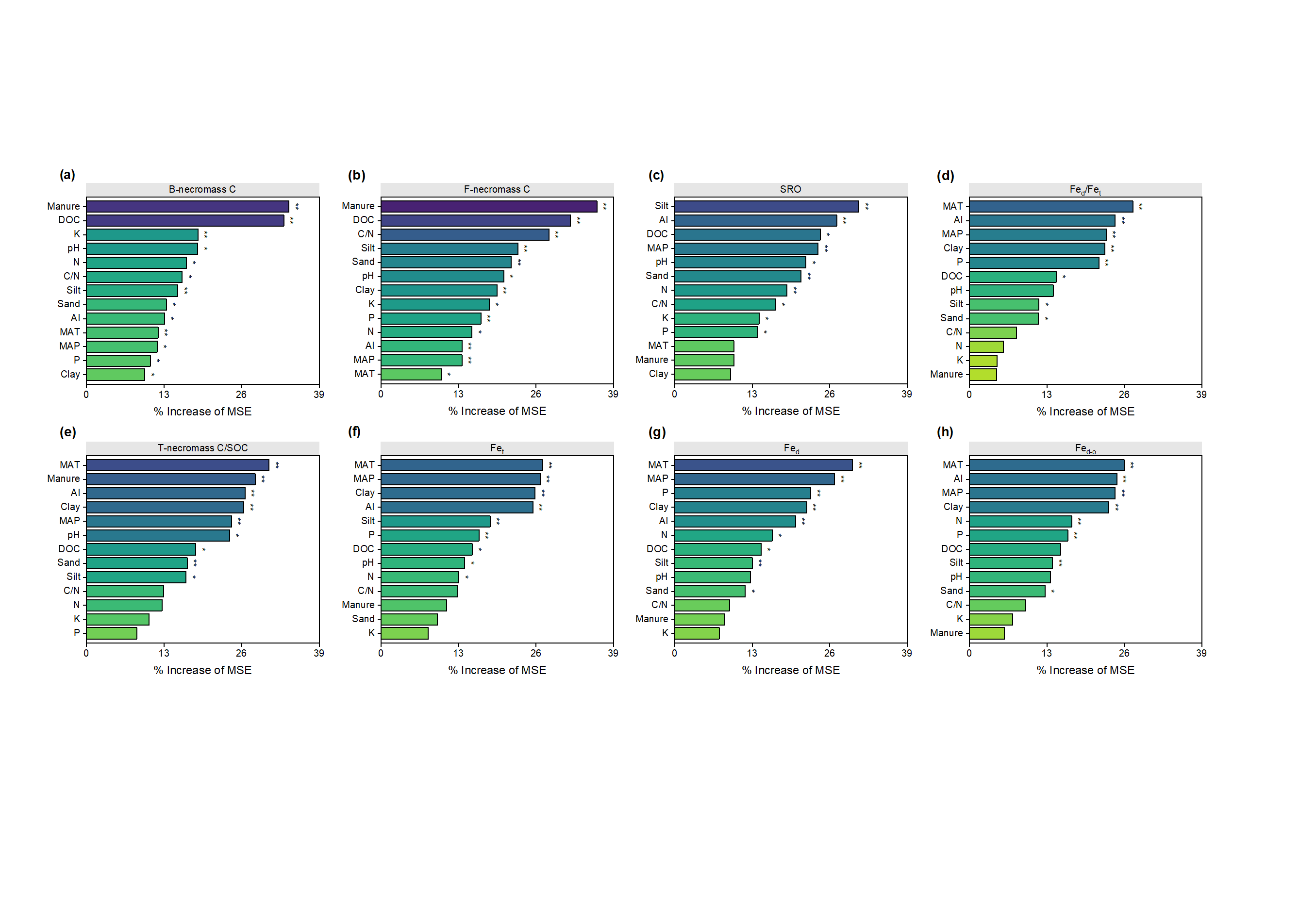


**Fig. S3. Relative importance of environmental drivers and fertilization history in predicting microbial necromass C and soil mineral properties.** Predictor importance is expressed as the percentage increase in mean squared error (%IncMSE) following permutation of each predictor, with higher values indicating greater importance in model prediction. B-necromass C, bacterial necromass C; F-necromass C, fungal necromass C. SRO, short-range-ordered minerals. Fe_t_, total Fe. Fe_d_, the dithionite-citrate-bicarbonate (DCB) extracted Fe minerals. T-necromass C/SOC, total necromass C/ soil organic carbon. Fe_d-o_, the difference between Fe_d_ and Fe_o_ subtracted. C/N: soil organic carbon/soil total nirtogen. DOC, soil dissolved organic carbon. N, nitrogen fertilizer rate. P, phosphorus fertilizer rate. K, potassium fertilizer rate. MAP, mean annual precipitation. MAT, mean annual temperature. AI, aridity index. Asterisks above bars indicate statistically significant variable importance based on permutation tests. **p* < 0.05, ***p* < 0.01. ns, not significant. N = 54.


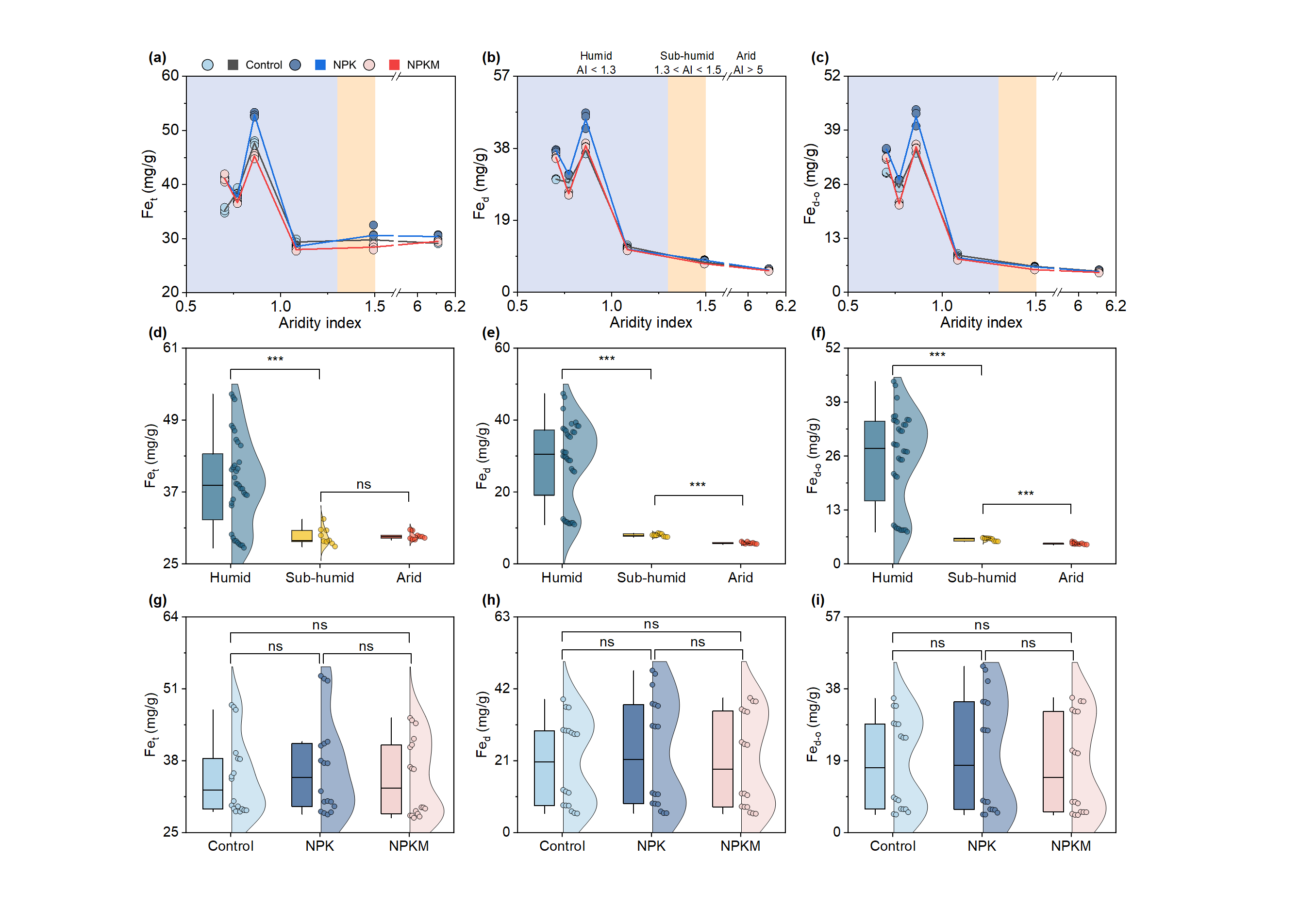


**Fig. S4. Changes in iron oxides with aridity index (AI) and fertilization regimes. a-c** Changes in iron minerals with AI. **d-i** Violin plots of the effects of AI and fertilization on minerals. All fertilized soils are divided into three groups based on aridity to reflect soil moisture conditions. S1-S4, including YT, JX, QY, SY, represent humid soils (AI < 1.3); S5 (GZL) represent sub-humid soils (1.3 < AI < 1.5); S6 (XJ) represent arid soils (AI > 5). Fe_t_, total Fe. Fe_d_, the dithionite-citrate-bicarbonate (DCB) extracted Fe minerals. Fe_d-o_, the difference between Fe_d_ and Fe_o_ subtracted. Control, no fertilizers; NPK, mineral fertilization with nitrogen, phosphorus, and potassium; NPKM, mineral fertilizer plus manure. AI, aridity index. ****p* < 0.001. ns, not significant. Data are means ± SE. The dataset for **(d-f)** includes N = 36 (4 soils including S1-S4), 9 (1 soil including S5) and 9 (1 soil including S6) for arid soils. N = 18 for each treatment in **(g-i)**.


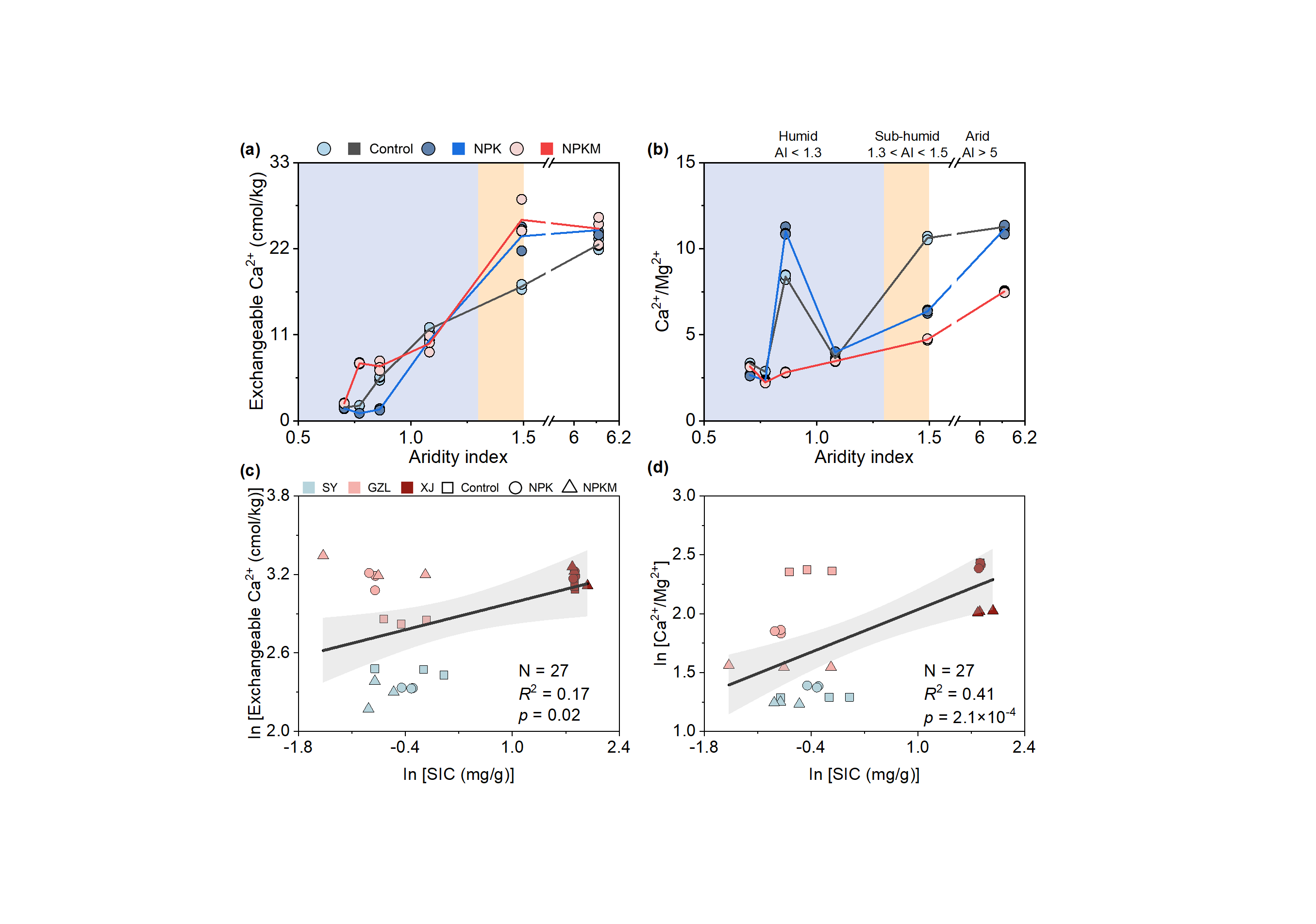


**Fig. S5. Changes in exchangeable elements with aridity index.** The solid lines indicate linear regressions, and the shaded areas represent 95 confidence intervals. AI, aridity index. SIC, soil inorganic carbon. Control, no fertilizers; NPK, mineral fertilization with nitrogen, phosphorus, and potassium; NPKM, mineral fertilizer plus manure. Different colors represent different sites and shapes represent fertilization treatments. N = 54.


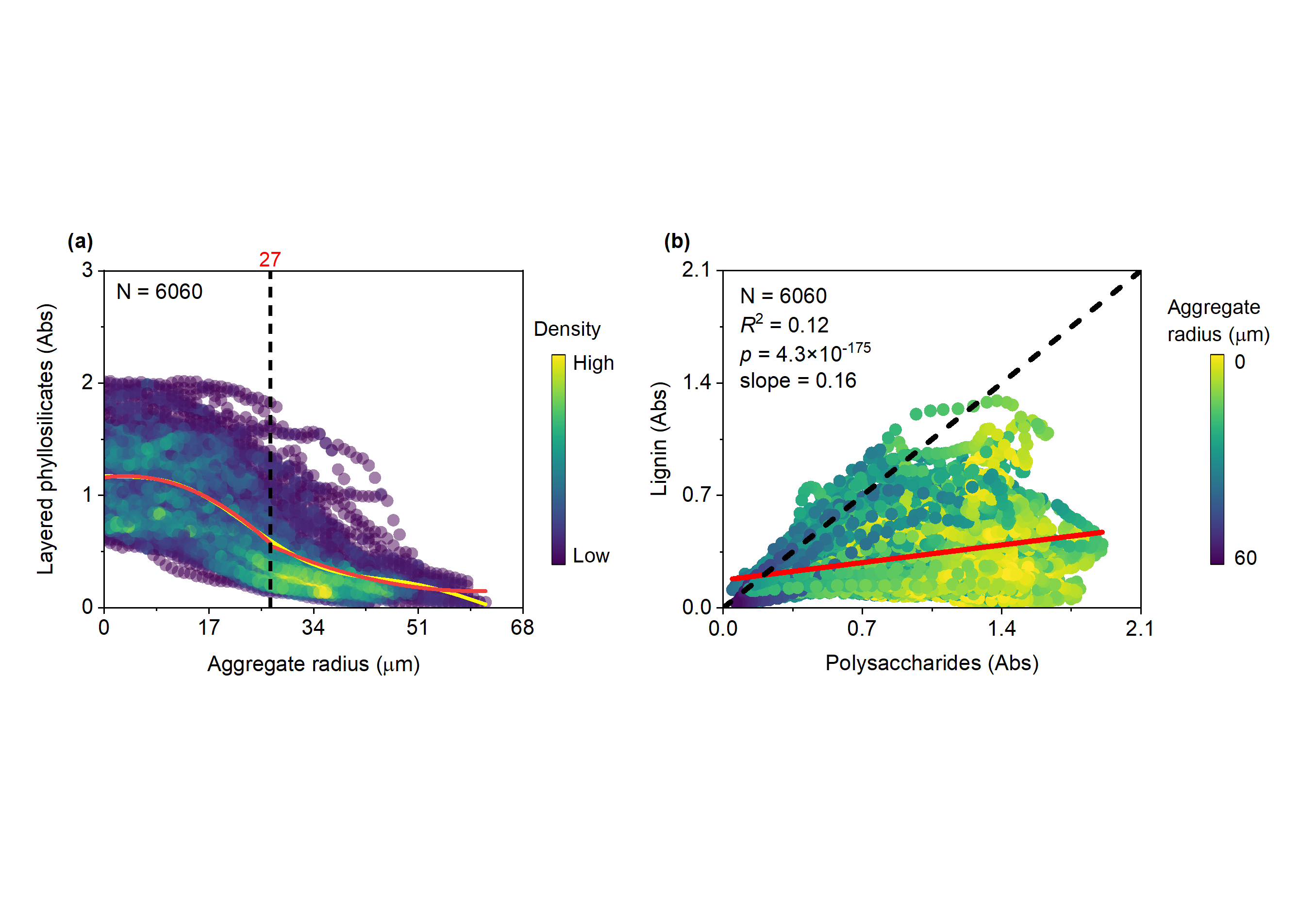


**Fig. S6. Changes in layered phyllosilicates with aggregate radius (a) and correlation between lignin and polysaccharides (b).** Key infrared absorption peaks include layered phyllosilicate (3,621 cm^−1^), lignin (1,512 cm^−1^), and polysaccharides (1,030 cm^−1^). The radius threshold of layered phyllosilicates is identified as 27 μm. Once this radius was reached, small increases in radius led to drastic decline in the layered phyllosilicates abundance.

**Table S1** Changes in soil pH and DOC in response to fertilization regimes across six long-term (27–38 years) fertilization sites ^a^.

| Soil indices | Sites | Control | NPK | NPKM |
| --- | --- | --- | --- | --- |
| pH | S1 | 4.71±0.02c | 4.90±0.03b | 5.01±0.03a |
|  | S2 | 4.92±0.03b | 4.71±0.02c | 5.90±0.03a |
|  | S3 | 5.39±0.02b | 4.20±0.02c | 6.11±0.02a |
|  | S4 | 6.02±0.01a | 5.28±0.03c | 5.57±0.02b |
|  | S5 | 7.60±0.01a | 6.54±0.04c | 7.11±0.02b |
|  | S6 | 8.33±0.02a | 7.83±0.02c | 8.26±0.02b |
| DOC (mg/kg) | S1 | 144.35±1.88c | 181.21±3.57b | 219.46±4.01a |
|  | S2 | 170.07±2.17b | 174.06±0.81b | 244.21±1.30a |
|  | S3 | 126.31±1.09c | 291.99±3.00b | 326.09±3.16a |
|  | S4 | 146.99±3.72c | 240.10±2.74b | 284.61±3.66a |
|  | S5 | 151.72±2.30c | 238.07±5.21b | 412.53±0.40a |
|  | S6 | 132.90±1.90b | 126.64±5.26b | 313.53±1.37a |

^a^ DOC, soil dissolved organic carbon. Control, no fertilizers; NPK, mineral fertilization with nitrogen, phosphorus, and potassium; NPKM, mineral fertilizer plus manure. The geographic locations of sites S1-S6 are shown in Fig. 1. Sites S1-S4 are classified as humid, whereas sites S5 and S6 are sub-humid and arid, respectively. Significant differences among fertilization treatments were determined using one-way ANOVA followed by Duncan’s multiple range test at *p* < 0.05. Data are means ± SE (n = 3).

**Table S2** Application rates of fertilizers used in RF models.

| Site | Treatment | Application rates of fertilizers (kg/ha) | | | NPK | Manure |
| --- | --- | --- | --- | --- | --- | --- |
|  |  | N | P | K | kg/ha | t/ha |
| S1 | Control | 0 | 0 | 0 | 0 | 0 |
|  | NPK | 120 | 32.8 | 62.5 | 215.3 | 0 |
|  | NPKM | 84 | 32.8 | 62.5 | 179.3 | 7.2 |
| S2 | Control | 0 | 0 | 0 | 0 | 0 |
|  | NPK | 120 | 26.2 | 100 | 246.2 | 0 |
|  | NPKM | 120 | 26.2 | 100 | 246.2 | 15 |
| S3 | Control | 0 | 0 | 0 | 0 | 0 |
|  | NPK | 300 | 52.4 | 100 | 452.4 | 0 |
|  | NPKM | 90 | 52.4 | 100 | 242.4 | 42 |
| S4 | Control | 0 | 0 | 0 | 0 | 0 |
|  | NPK | 120 | 26.2 | 50 | 196.2 | 0 |
|  | NPKM | 120 | 26.2 | 50 | 196.2 | 27 |
| S5 | Control | 0 | 0 | 0 | 0 | 0 |
|  | NPK | 165 | 36 | 68.8 | 269.8 | 0 |
|  | NPKM | 50 | 36 | 68.8 | 154.8 | 34.5 |
| S6 | Control | 0 | 0 | 0 | 0 | 0 |
|  | NPK | 241.5 | 60.2 | 50.4 | 352.1 | 0 |
|  | NPKM | 84.9 | 22.4 | 10.1 | 117.4 | 30 |

**Table S3** Nutrient content of manure across the six long-term experimental sites ^a^.

| Site | N | P | K |
| --- | --- | --- | --- |
|  | g/kg | | |
| S1 | 36.5 | 23.0 | 52.0 |
| S2 | 12.0 | 9.0 | 10.0 |
| S3 | 18.2 | 13.7 | 13.3 |
| S4 | 5.8 | 3.6 | 9.0 |
| S5 | 3.5 | 1.3 | 3.4 |
| S6 | 8.0 | 2.3 | 3.0 |

^a^ Data for manure nutrient content at S1-S6 are derived from references (Cai et al., 2014; Ling et al., 2014; Liu, 2019; Wang, Tang, Zhang, Schroder, & He, 2014; Xie et al., 2015; Xu et al., 2016)

**Table S4** Variations in microbial necromass C for NPK and NPKM treatments compared to Control (%) ^a^.

| **Site** | **AI** | **B-necromass C** | | **F-necromass C** | | **F/B-necromass C** | |
| --- | --- | --- | --- | --- | --- | --- | --- |
|  | **/** | **NPK** | **NPKM** | **NPK** | **NPKM** | **NPK** | **NPKM** |
| S1 | 0.70 | 14.1 ~ 46.7 | 24.5 ~ 69.0 | 3.7 ~ 33.4 | 18.8 ~ 64.3 | -22.6 ~ 9.7 | -23.2 ~ 18.9 |
| S2 | 0.77 | -5.1 ~ 44.1 | 35.7 ~ 92.2 | -3.1 ~ 39.9 | 40.2 ~ 101.5 | -24.0 ~ 31.1 | -17.5 ~ 33.9 |
| S3 | 0.86 | 2.5 ~ 57.6 | 47.6 ~ 127.2 | 19.6 ~ 53.8 | 148.9 ~ 225.3 | -23.6 ~ 49.0 | 9.8 ~ 115.4 |
| S4 | 1.08 | -19.0 ~ 44.8 | 3.7 ~ 88.4 | -9.2 ~ 10.5 | 2.2 ~ 22.5 | -33.5 ~ 32.4 | -42.4 ~ 10.8 |
| S5 | 1.49 | -11.6 ~ 62.6 | 132.1 ~ 282.3 | -1.6 ~ 8.1 | 76.6 ~ 106.8 | -39.5 ~ 22.2 | -53.8 ~ -17.3 |
| S6 | 6.11 | 4.4 ~ 69.0 | 329.0 ~ 525.2 | -14.7 ~ 18.8 | 45.3 ~ 99.3 | -43.5 ~ -4.4 | -75.1 ~ -55.6 |

^a^ B-necromass C, bacterial necromass C. F-necromass C, fungal necromass C. F/B-necromass C, fungal/bacterial necromass C. Control, no fertilizers; NPK, mineral fertilization with nitrogen, phosphorus, and potassium; NPKM, mineral fertilizer plus manure. AI, aridity index.

**Table S5** Effects of fertilization and aridity index on microbial necromass C and minerals ^a^.

| Parameters | F value | | |
| --- | --- | --- | --- |
|  | Fertilization | Aridity index | Fertilization × Aridity index (Interaction) |
| B-necromass C | 155.136*** | 25.709*** | 26.751*** |
| F-necromass C | 27.153*** | 17.424*** | 1.115 |
| SRO | 1.177 | 54.922*** | 1.126 |
| Fe_d_/Fe_t_ | 0.044 | 50.391*** | 0.048 |
| T-necromass/SOC | 0.06 | 39.250*** | 0.284 |
| Fe_t_ | 0.252 | 10.952*** | 0.033 |
| Fe_d_ | 0.102 | 29.888*** | 0.091 |
| Fe_d-o_ | 0.084 | 24.996*** | 0.095 |

^a^ *p* values represent statistical significance, based on two-way ANOVAs. Significant effects are indicated as ****p* < 0.001.

**Table S6** Simple effects of B-necromass C under different aridity index and fertilization treatments ^a^.

| Fixed factor | Pairwise comparison | Mean difference | SE | *t* value | *p* value |
| --- | --- | --- | --- | --- | --- |
| Control | Humid - Sub-humid | -0.118 | 0.178 | -0.663 | 1.000 |
|  | Humid - Arid | 0.559 | 0.178 | 3.136 | 0.009 |
|  | Sub-humid - Arid | 0.677 | 0.225 | 3.004 | 0.013 |
| NPK | Humid - Sub-humid | -0.141 | 0.178 | -0.792 | 1.000 |
|  | Humid - Arid | 0.581 | 0.178 | 3.264 | 0.006 |
|  | Sub-humid - Arid | 0.722 | 0.225 | 3.207 | 0.007 |
| NPKM | Humid - Sub-humid | -1.909 | 0.178 | -10.719 | <0.001 |
|  | Humid- Arid | -1.145 | 0.178 | -6.429 | <0.001 |
|  | Sub-humid - Arid | 0.764 | 0.225 | 3.392 | 0.004 |
| Humid | Control - NPK | -0.223 | 0.113 | -1.979 | 0.162 |
|  | Control - NPKM | -0.654 | 0.113 | -5.807 | <0.001 |
|  | NPK - NPKM | -0.431 | 0.113 | -3.828 | 0.001 |
| Sub-humid | Control - NPK | -0.246 | 0.225 | -1.091 | 0.843 |
|  | Control - NPKM | -2.445 | 0.225 | -10.853 | <0.001 |
|  | NPK - NPKM | -2.199 | 0.225 | -9.762 | <0.001 |
| Arid | Control - NPK | -0.2 | 0.225 | -0.888 | 1.000 |
|  | Control - NPKM | -2.357 | 0.225 | -10.465 | <0.001 |
|  | NPK - NPKM | -2.157 | 0.225 | -9.577 | <0.001 |

^a^ Simple effects were examined using pairwise comparisons of estimated marginal means following a significant aridity index × fertilization interaction. Differences in mean represent the subtraction of the second level from the first level listed. Standard errors (SE) and t values are shown. *p* values were adjusted for multiple comparisons using the Bonferroni correction. Significant differences are indicated at *p* < 0.05.

**Table S7** Post hoc pairwise comparisons for significant main effects detected by two-way ANOVA ^a^.

| Variable | Factor | Pairwise comparison | Mean difference | SE | *t* value | *p* value |
| --- | --- | --- | --- | --- | --- | --- |
| F-necromass C | Fertilization | Control - NPK | -0.207 | 0.348 | -0.595 | 1.000 |
|  |  | Control - NPKM | -2.319 | 0.348 | -6.659 | <0.001 |
|  |  | NPK - NPKM | -2.112 | 0.348 | -6.063 | <0.001 |
|  | Aridity index | Humid - Sub-humid | -0.143 | 0.318 | -0.451 | 1.000 |
|  |  | Humid - Arid | 1.805 | 0.318 | 5.677 | <0.001 |
|  |  | Sub-humid - Arid | 1.948 | 0.402 | 4.844 | <0.001 |
| SRO | Aridity index | Humid - Sub-humid | 0.54 | 0.223 | 2.417 | 0.059 |
|  |  | Humid - Arid | 2.338 | 0.223 | 10.475 | <0.001 |
|  |  | Sub-humid - Arid | 1.799 | 0.282 | 6.37 | <0.001 |
| Fe_d_/Fe_t_ | Aridity index | Humid - Sub-humid | 0.448 | 0.063 | 7.151 | <0.001 |
|  |  | Humid - Arid | 0.522 | 0.063 | 8.334 | <0.001 |
|  |  | Sub-humid - Arid | 0.074 | 0.079 | 0.935 | 1.000 |
| T-necromass/SOC | Aridity index | Humid - Sub-humid | 19.527 | 2.932 | 6.661 | <0.001 |
|  |  | Humid - Arid | 20.688 | 2.932 | 7.057 | <0.001 |
|  |  | Sub-humid - Arid | 1.16 | 3.708 | 0.313 | 1.000 |
| Fe_t_ | Aridity index | Humid - Sub-humid | 8.926 | 2.453 | 3.639 | 0.002 |
|  |  | Humid - Arid | 8.857 | 2.453 | 3.611 | 0.002 |
|  |  | Sub-humid - Arid | -0.069 | 3.102 | -0.022 | 1.000 |
| Fe_d_ | Aridity index | Humid - Sub-humid | 20.787 | 3.66 | 5.68 | <0.001 |
|  |  | Humid - Arid | 22.965 | 3.66 | 6.275 | <0.001 |
|  |  | Sub-humid - Arid | 2.178 | 4.629 | 0.47 | 1.000 |
| Fe_d-o_ | Aridity index | Humid - Sub-humid | 19.558 | 3.664 | 5.338 | <0.001 |
|  |  | Humid - Arid | 20.559 | 3.664 | 5.611 | <0.001 |
|  |  | Sub-humid - Arid | 1 | 4.635 | 0.216 | 1.000 |

^a^ Post hoc pairwise comparisons were conducted only for factors showing significant main effects in the two-way ANOVA without significant interaction effects. Comparisons were based on estimated marginal means, and p values were adjusted using the Bonferroni correction.

**Table S8** Best models for each variable.

| **Variable** | **Group** | **Linear AICc** | **Quadratic AICc** | **GAM AICc** | **AICc of threshold models ^a^** | | | | | |
| --- | --- | --- | --- | --- | --- | --- | --- | --- | --- | --- |
|  |  |  |  |  | **Segmented** | **Step** | **Stegmented** | **M12** | **M21** | **M22** |
| Amides | All | -1379.9 | -1531.2 | -1609.9 | -1594.1 | -1523.1 | -1595.4 | **-18809.2** | -18798.3 | -18807.5 |
| Layered phyllosilicates | All | 4620.2 | 4620.0 | 4372.0 | 4556.4 | 4463.0 | 4464.3 | -12815.1 | -12820.2 | **-12824.1** |
| Polysaccharides | All | 2727.4 | 2706.0 | 2397.7 | 2591.5 | 2534.7 | 2534.4 | -14788.7 | -14785.2 | **-14797.0** |
| Humification index | Humid | 154.7 | 154.9 | 37.5 | 136.2 | 85.4 | 87.1 | -11530.0 | -11553.4 | **-11555.2** |
|  | Sub-humid | 398.6 | 399.1 | 397.6 | 400.0 | 393.3 | 392.8 | -2392.3 | -2390.8 | **-2392.5** |
|  | Arid | -326.8 | -394.2 | -507.1 | -478.4 | -445.0 | -480.8 | -3296.5 | **-3306.4** | -3305.4 |
|  | Control | -33.1 | -31.1 | -91.5 | -36.7 | -63.2 | -61.2 | -5992.9 | -6004.3 | **-6016.5** |
|  | NPK | -224.8 | -223.3 | -248.3 | -227.0 | -230.1 | -233.8 | -5826.0 | -5823.1 | **-5828.7** |
|  | NPKM | 861.9 | 832.6 | 718.5 | 759.4 | 779.4 | 757.2 | -4957.5 | **-4959.8** | -4958.6 |

^a^ Both linear and nonlinear [quadratic and generalized additive models (GAMs)] regressions were used to the relationships between variables with microbial necromass and functional groups. Lower AICc values indicate a better fit of the model. The existence of thresholds can be explored and nonlinear trends determined only when the nonlinear model is suitable. Then we fitted step (a linear regression that modifies only intercept at a given aridity level), segmented (showing changes both in intercept and slope at a given aridity level) regressions, segmented (exhibiting changes only in slope at a given aridity level) regressions, M12 (linear-quadratic), M21 (quadratic-linear) and M22 (quadratic-quadratic). Characteristic absorption bands were assigned as follows: 3,621 cm^−1^ (structural O–H in layered phyllosilicates), 1,643 cm^−1^ (N–H bending in amides), and 1,030 cm^−1^ (C–OH in polysaccharides). To evaluate the degree of organic matter degradation, we calculated a spatially resolved humification index (HI), defined as the ratio of absorbance at 1,643 cm⁻¹ (amides) to 1,030 cm⁻¹ (polysaccharides), using synchrotron infrared (IR) spectromicroscopy.

**References**

Cai, Z., Wang, B., Xu, M., Zhang, H., He, X., Zhang, L., & Gao, S. (2014). Intensified soil acidification from chemical N fertilization and prevention by manure in an 18-year field experiment in the red soil of southern China. *Journal of Soils and Sediments, 15*(2), 260–270. doi:10.1007/s11368-014-0989-y

Chen, Y., Sun, R., Sun, T., Liang, Y., Jiang, Y., & Sun, B. (2018). Organic amendments shift the phosphorus-correlated microbial co-occurrence pattern in the peanut rhizosphere network during long-term fertilization regimes. *Applied Soil Ecology, 124*, 229–239. doi:10.1016/j.apsoil.2017.11.023

Dou, X., He, P., Cheng, X., & Zhou, W. (2016). Long-term fertilization alters chemically-separated soil organic carbon pools: Based on stable C isotope analyses. *Scientific Reports, 6*(1), 19061. doi:10.1038/srep19061

Hua, W., Luo, P., An, N., Cai, F., Zhang, S., Chen, K., . . . Han, X. (2020). Manure application increased crop yields by promoting nitrogen use efficiency in the soils of 40-year soybean-maize rotation. *Scientific Reports, 10*(1), 14882. doi:10.1038/s41598-020-71932-9

Huang, S., Peng, X., Huang, Q., & Zhang, W. (2010). Soil aggregation and organic carbon fractions affected by long-term fertilization in a red soil of subtropical China. *Geoderma, 154*(3-4), 364–369. doi:10.1016/j.geoderma.2009.11.009

Lal, R. (2004). Soil carbon sequestration impacts on global climate change and food security. *Science, 304*(5677), 1623–1627. doi:10.1126/science.1097396

Ling, N., Sun, Y., Ma, J., Guo, J., Zhu, P., Peng, C., . . . Shen, Q. (2014). Response of the bacterial diversity and soil enzyme activity in particle-size fractions of Mollisol after different fertilization in a long-term experiment. *Biology and Fertility of Soils, 50*(6), 901–911. doi:10.1007/s00374-014-0911-1

Liu, K. (2019). *Mechanism of pH and organic carbon levels influencing aggregate-associated potassium distribution in red soil under long-term fertilization.* (Doctoral dissertation), Chinese Academy of Agricultural Sciences, Beijing, China.

Liu, K., Hu, Z., Ye, H., Li, D., Huang, Q., Yu, X., . . . Wang, S. (2016). Long-term fertilization changes soil nitrogen and phosphorus activation in red soil under double maize system. *Journal of Soil and Water Conservation, 30*(2), 188–207. doi:10.13870/j.cnki.stbcxb.2016.02.033

Luo, P., Han, X., Wang, Y., Han, M., Shi, H., Liu, N., & Bai, H. (2015). Influence of long-term fertilization on soil microbial biomass, dehydrogenase activity, and bacterial and fungal community structure in a brown soil of northeast China. *Annals of Microbiology, 65*(1), 533–542. doi:10.1007/s13213-014-0889-9

Song, Z., Gao, H., Zhu, P., Peng, C., Deng, A., Zheng, C., . . . Zhang, W. (2015). Organic amendments increase corn yield by enhancing soil resilience to climate change. *The Crop Journal, 3*(2), 110–117. doi:10.1016/j.cj.2015.01.004

Wang, Y., Tang, J., Zhang, H., Schroder, J. L., & He, Y. (2014). Phosphorus Availability and Sorption as Affected by Long‐Term Fertilization. *Agronomy Journal, 106*(5), 1583–1592. doi:10.2134/agronj14.0059

Xie, H., Li, J., Zhang, B., Wang, L., Wang, J., He, H., & Zhang, X. (2015). Long-term manure amendments reduced soil aggregate stability via redistribution of the glomalin-related soil protein in macroaggregates. *Scientific Reports, 5*, 14687. doi:10.1038/srep14687

Xu, Y., Liu, H., & Wang, X. (2016). Evolution of soil organic carbon and crop yield under long-term fertilization in grey desert soils. *Chinese Journal of Eco-Agriculture, 24*(2), 154–162. doi:10.13930/j.cnki.cjea.151082

Yu, G.-H., & Liu, S. (2022). Visualizing mineral-associated organic matters in long-term fertilization treated soils by NanoSIMS and SR-FTIR. *Frontiers in Soil Science, 2*, 847623. doi:10.3389/fsoil.2022.847623

Yu, G., Xiao, J., Hu, S., Polizzotto, M., Zhao, F., McGrath, S., . . . Shen, Q. (2017). Mineral availability as a key regulator of soil carbon storage. *Environmental Science and Technology, 51*(9), 4960–4969. doi:10.1021/acs.est.7b00305

Zhang, H.-M., Wang, B.-R., Xu, M.-G., & Fan, T.-L. (2009). Crop yield and soil responses to long-term fertilization on a red soil in Southern China. *Pedosphere, 19*, 199–207. doi:10.1016/S1002-0160(09)60109-0
